# Assessment of the Efficacy and Mode of Action of Benzo(1,2,3)-Thiadiazole-7-Carbothioic Acid S-Methyl Ester (BTH) and Its Derivatives in Plant Protection Against Viral Disease

**DOI:** 10.3390/ijms20071598

**Published:** 2019-03-30

**Authors:** Patryk Frąckowiak, Henryk Pospieszny, Marcin Smiglak, Aleksandra Obrępalska-Stęplowska

**Affiliations:** 1 Department of Molecular Biology and Biotechnology, Institute of Plant Protection, National Research Institute, ul. Władysława Węgorka 20, 60-318 Poznań, Poland; p.frackowiak@iorpib.poznan.pl; 2 Department of Virology and Bacteriology, Institute of Plant Protection, National Research Institute, ul. Władysława Węgorka 20, 60-318 Poznań, Poland; h.pospieszny@iorpib.poznan.pl; 3 Poznań Science and Technology Park, Adam Mickiewicz University Foundation, ul. Rubież 46, 61-612 Poznań, Poland; marcin.smiglak@gmail.com

**Keywords:** BTH, ionic liquids, systemic acquired resistance (SAR), signalling pathway, resistance inducers, SA, JA, ET, plant virus, plant-virus interaction, viral disease

## Abstract

Systemic acquired resistance (SAR) induction is one of the primary defence mechanisms of plants against a broad range of pathogens. It can be induced by infectious agents or by synthetic molecules, such as benzo(1,2,3)-thiadiazole-7-carbothioic acid S-methyl ester (BTH). SAR induction is associated with increases in salicylic acid (SA) accumulation and expression of defence marker genes (e.g., phenylalanine ammonia-lyase *(PAL)*, the pathogenesis-related *(PR)* protein family, and non-expressor of PR genes *(NPR1)*). Various types of pathogens and pests induce plant responses by activating signalling pathways associated with SA, jasmonic acid (JA) and ethylene (ET). This work presents an analysis of the influence of BTH and its derivatives as resistance inducers in healthy and virus-infected plants by determining the expression levels of selected resistance markers associated with the SA, JA, and ET pathways. The phytotoxic effects of these compounds and their influence on the course of viral infection were also studied. Based on the results obtained, the best-performing BTH derivatives and their optimal concentration for plant performance were selected, and their mode of action was suggested. It was shown that application of BTH and its derivatives induces increased expression of marker genes of both the SA- and JA-mediated pathways.

## 1. Introduction

Plants are organisms with limited mobility, which determines their ability to defend against pathogens and pests. Thus, in response to infection or insect foraging, changes in the expression of genes associated with stress and defence are induced in plants [1]; however, the plant resistance response depends on the type of pathogen, environmental conditions [2], and plant species infected [3]. Among such pathogen attacks, viral infections are one of the most difficult to control because there is no universal remedy for virus-induced diseases. After overcoming the mechanical barrier that prevents viral penetration into the plant tissues, the infection may spread very quickly, leading to disease that will result in either recovery or the death of the host. The approaches undertaken to reduce viral diseases in crops include the breeding of resistant varieties, the elimination of vectors carrying these pathogens and the elimination of infected plants to avoid further spread of the disease. In 1901, it was discovered that plants that were previously infected by pathogens could better resist further infection [4]. From the beginning of their existence, plant hosts have evolved diverse mechanisms allowing them to respond to different types of infectious agents, such as specific recognition of pathogen-derived molecules by the inducible immune system [5,6,7,8,9].

Several types of induced resistance have been described in plants: localized acquired resistance (LAR) [10], induced systemic resistance (ISR) [11,12,13,14,15,16], ISR reliant on β-aminobutyric acid, and systemic acquired resistance (SAR) [10,17,18,19,20,21,22,23]. LAR was observed and named by Ross, who discovered areas immune to viral infection (the LAR zone) near tobacco mosaic virus (TMV)-induced local lesions on Samsun NN tobacco leaves [24]. LAR and the hypersensitive response (HR) in plants are the first rapid local responses to the appearance of a pathogen. Ross also observed that, beyond the LAR zone, SAR is induced in other uninoculated leaves. Many studies have shown that, in tissues where SAR was activated, a subset of defence responses were induced locally, such as the oxidative burst [25]. In comparison to the locally induced LAR, SAR and ISR are systemically induced. The main features differentiating these two types of systemic resistance are the nature of the elicitor and the regulatory signalling pathways involved in a SAR or ISR response. SAR is a highly desirable form of resistance in plants, induced by a large spectrum of pathogens in distal, uninfected plant tissues, wherein accumulation of pathogenesis-related proteins (*PR1*, chitinase, glucanase), and salicylic acid (SA) takes place [12]. In contrast, ISR is considered as induced by factors such as plant growth-promoting rhizobacteria (PGPR) or pests feeding on the surface of the leaf blade and is associated with JA and ET pathway induction [12].

SAR provides long-term resistance (in some cases up to several months) [26,27]. Its elicitors are usually molecules originating from bacteria, pathogenic fungi, and viruses, such as proteins, complex polysaccharides, and SAR regulators such as methyl salicylic acid (MeSA), azelaic acid (AzA), dihydroabetinal (DA) glycerol-3-phosphate (G3P), and pipecolic acid (PIP) and its derivatives [23,27,28]. Therefore, one alternative approach to induce plant immune mechanisms may be the use of natural or synthetic resistance-inducing substances. Induction of SAR before contact with pathogens gives plants the opportunity to be more efficient and effective in pathogen defence. One of the first compounds developed to be capable of activating systemic resistance was 2,6-dichlorosionicotinic acid and its methyl ester (both referred to as INA), but they were insufficiently tolerated by some crops, causing phytotoxic effects in plants [26].

In 1996, a new chemical compound that induced SAR called benzo(1,2,3)-thiadiazole-7-carbothioic acid S-methyl ester (BTH) was synthesized and described [29]. There is evidence that BTH activates resistance in various plant species against a large spectrum of pathogens, such as viruses, (e.g., TMV in tobacco [29,30,31,32,33]), fungi and bacteria, such as *Cercospora nicotianae* or *Pseudomonas syringae pv.* tabaci [30,34]. BTH can also prime defence reactions in plants [7]. Due to its positive effects on SAR induction, many BTH derivatives were then obtained. Kunz and colleagues considered and synthesized a number of different modifications of the chemical structure of BTH [35], developing the derivative used in a commercial BTH-BION (CGA 245704), which was the one of the most effective related compounds based on its biological properties and structural activity. However, in some cases, the BION preparation was not properly used, and it was found to be phytotoxic at overly high concentrations [36]. To limit or eliminate this phytotoxicity without limiting the beneficial induction of plant resistance, many other derivatives of BTH have been created [37,38]. Interest in the production of synthetic chemicals that would be able to mimic natural SAR inducers has increased in recent decades.

SAR induction in plants is strongly linked to the synthesis of salicylic acid (SA), especially in distal tissues, in response to pathogen attack [21]. SA is a small phenolic compound that not only participates in the induction of immunity but also regulates a number of aspects of plant maturation, including thermogenesis, growth, respiration, and thermotolerance [39]. In addition to SA, jasmonic acid (JA) and ethylene (ET) also participate in plant resistance, but they are synthesized to a greater extent in response to tissue damage caused by insects and during the induction of ISR [15,40,41]. A growing body of literature reports that the SA and JA/ET pathways do not function independently. Rather, they are involved in a complex signalling network in which the different pathways influence each other through both positive [42,43] and negative [44] regulatory interactions.

To better understand the functioning of resistance inducers and their potential to induce SAR, differences in the expression of specific resistance marker genes associated with the SA pathway, among others, have been assessed [45,46]. The most commonly tested genes belong to: (a) the family of pathogenesis-related protein (*PR*) genes (including the *PR1* gene, glucan endo-1,3-beta-glucanase (*GluB* or *PR2*)); (b) the phenylalanine ammonia-lyase gene (*PAL*); and (c) non-expressor of pathogenesis-related genes 1 (*NPR1*). The *PAL* gene encodes an enzyme associated with phenylalanine modification in the phenylpropanoid pathway and is involved with regulation of SA production during SAR induction [47]. SA accumulation activates the expression of *NPR1*, which is transferred from the cytoplasm to the nucleus and interacts with TGA (a basic leucine zipper transcription factor) to induce downstream expression of *PR* genes (*PR1, PR2, PR5*) [48]. The mode of action of resistance inducers is complex. Therefore, the levels of accumulation of other secondary metabolites, including JA and ethylene, have also been examined in these studies [45]. Among the marker genes of the ethylene pathway, the ethylene receptor (*ER*) and a serine/threonine-protein kinase (*CTR*) are frequently studied; in turn, the JA pathway is jasmonic acid-amido synthetase 1 (*JAR1*), lipoxygenase (*LOX*), and proteinase inhibitor II (*PI II*). *ER* is a membrane-anchored receptor involved in ethylene signalling, while *CTR* is an ER-associated protein kinase (MAPKKK) that negatively regulates ethylene signalling and can lead to HR-type responses or programmed cell death (PCD) [49]. *LOX* is an enzyme that regulates JA production by transforming linoleic acid into oxophytodienoic acid (OPDA). *JAR1* is involved in the regulation of plant defensin gene expression [50], and *PI II* is a proteinase inhibitor that is inducible in leaves in response to herbivore attacks or other types of wounding [51,52].

Tomato mosaic virus (ToMV) is a serious plant pathogen belonging to the *Tobamovirus* genus in the *Virgaviridae* family. It has a non-segmented, positive-sense, single-stranded RNA genome ((+)ssRNA) of approximately 6400 nts, which encodes four proteins: two replication proteins (130K and 180K proteins), a movement protein (MP), and a coat protein (CP) [53,54]. Similar to TMV, another member of the *Tobamovirus* genus, ToMV infects tobacco plants locally (e.g., cv. Xanthi) causing necrotic spots on infected leaves, or systemically (e.g., cv. Samsun) causing mosaic symptoms, which are characterized by intermingled light and dark green regions. The time of virus incubation and spread in plant tissues makes this virus a good experimental model to test the potential for SAR induction by various substances and to study systemic viral RNA transport and accumulation in *N. tabacum* leaves [55].

**Figure 1:**
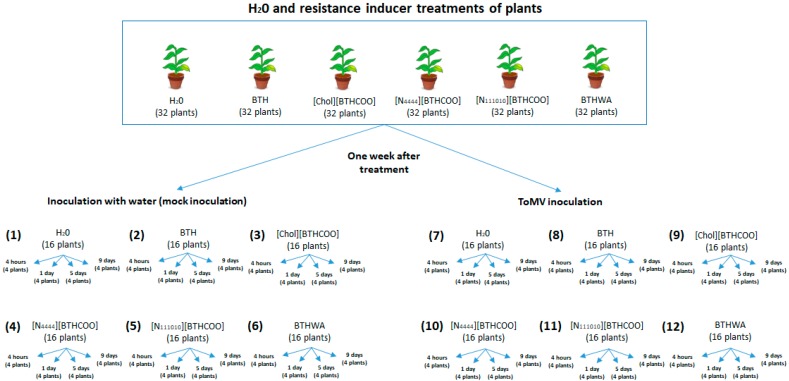
Diagram showing the experimental setup.

In this study, we analysed the efficacy and mode of action of BTH and BTH-derived resistance inducers, including an unpublished inducer, amide derivative of benzo(1,2,3)-thiadiazole-7-carbothioic acid (BTHWA) (Figure 1). Specifically, we: (a) estimated the phytotoxicity of chosen BTH derivatives; (b) evaluated the level of suppression of viral infection and transmission efficiency within the host upon resistance inducer application; (c) determined the levels of resistance induction on the basis of specific marker gene expression and assessed the relationship between signalling pathways linked to SA and JA/ET and the expression levels of selected genes; and (d) compared the resistance-related gene expression with symptoms of infection, accumulation of viral RNA in leaves and efficiency of pathogen transport within plants.

## 2. Results

### 2.1. Phytotoxicity of BTH and Its Derivatives after Soil Application

**Figure 2:**
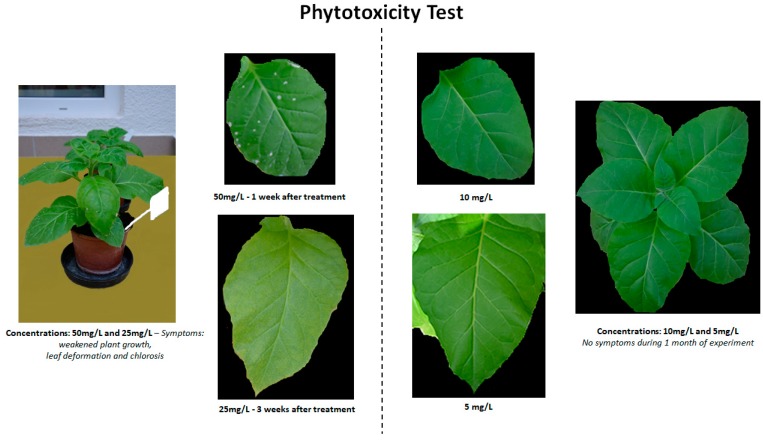
Example of phytotoxic symptoms observed after plants were treated with benzo(1,2,3)-thiadiazole-7-carbothioic acid S-methyl ester (BTH) at specific concentrations.

BTH, [Chol][BTHCOO], BTHWA, [N_4444_][BTHCOO], and [N_111010_][BTHCOO] were tested to evaluate their phytotoxicity to tobacco plants. Plants were grown in soil and watered with four concentrations (5, 10, 25, 50 mg/L) of BTH and its derivatives (100 mL per single application). Three plants were tested at each concentration. Resistance inducer-treated plants were observed for one month. The first phytotoxic effect on the treated plants was observed one week after inducer treatment in the plants treated with all inducers at a concentration of 50 mg/L (Figure 2). The effect was manifested by weakened plant growth, leaf deformation and chlorotic spots. The same situation was observed for plants treated with 25 mg/L at three weeks after treatment with all the tested inducers (Table 1). For the concentration of 10 mg/L, no negative effects were observed during the month of observation; therefore, this concentration was considered optimal and selected for further analysis.

### 2.2. Symptoms of Viral Infection in the Absence and Presence of Resistance Inducers

**Figure 3:**
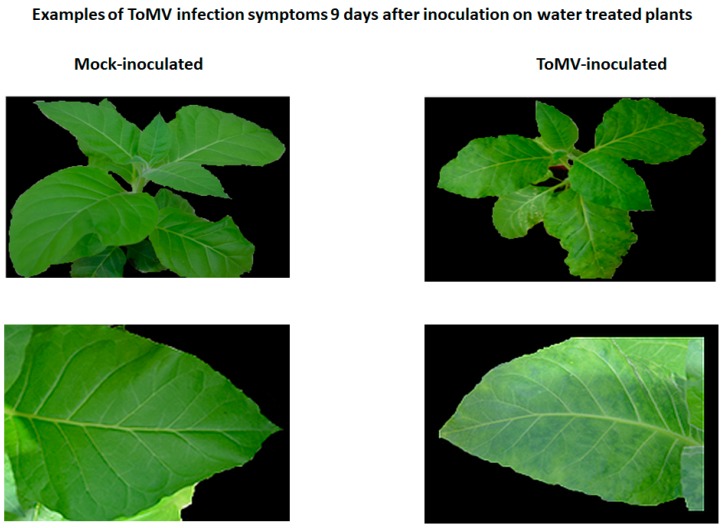
Healthy (mock-inoculated) plants (left) and tomato mosaic virus-induced symptoms (right) on plants not treated with resistance inducer, 9 days post virus inoculation.

After the experimental setup as presented in Figure 1, the first symptoms of viral disease (leaf mosaic) in plants were observed on the fifth day after virus inoculation and appeared solely on plants that were not treated with resistance inducers (Figure 1, Plant set 7). Nine days after inoculation, the disease symptoms were present throughout the non-induced virus-infected plants (Figure 3). Notably, the ToMV-infected plants treated with BTH and its derivatives (10 mg/L) grew normally and did not show any signs of infection during the entire 16-day experiment.

### 2.3. Analysis of the Expression of Defence-Related Marker Genes after BTH and Its Derivatives Treatments in the Absence of Pathogens

Each of the tested resistance inducers was associated with changes in the expression levels of resistance-related genes. Samples from non-infected plants were treated with inducers (Figure 1, “Mock-inoculated plants” from plant sets 1–6) were collected and prepared for analyses of resistance gene expression levels at each time point after inoculation (Table S1). The plants were pre-treated with BTH and its derivatives as mentioned above. After one week, the same plants were mock-inoculated or virus-inoculated and then harvested at four time points: 4 h post-inoculation (hpi), 1 day post-inoculation (dpi), 5 dpi and 9 dpi. Therefore, the times presented in the results are calculated from the time of mock inoculation.

**Figure 4:**
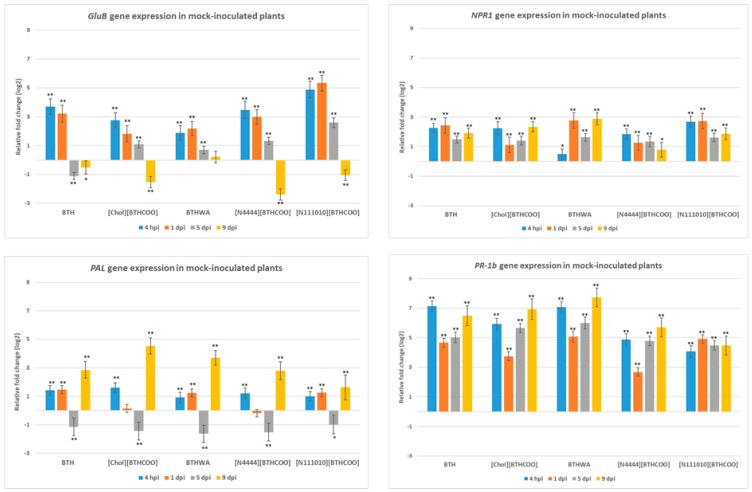
Relative changes in the expression of four systemic acquired resistance (SAR) marker genes (*GluB*—glucan endo-1,3-beta-glucanase; *NPR1*—non-expressor of pathogenic-related genes 1; *PAL*—phenylalanine ammonia-lyase gene; *PR-1b*—pathogenesis-related 1b) between mock-treated (Plant set 1) and inducer-treated plants (Plant sets 2–6). BTH, benzo(1,2,3)-thiadiazole-7-carbothioic acid S-methyl ester; [Chol][BTHCOO], cholinium benzo(1,2,3)-thiadiazole-7-carboxylate; BTHWA, amide derivative of benzo(1,2,3)-thiadiazole-7-carbothioic acid; [N_4444_][BTHCOO], tetrabutylammonium benzo(1,2,3)-thiadiazole-7-carboxylate; [N_111010_][BTHCOO], didecyldimethylammonium benzo(1,2,3)-thiadiazole-7-carboxylate. Each bar represents differential gene expression on the log2 scale at different time points: 4 h post inoculation (hpi), 1 day post inoculation (dpi), 5 dpi and 9 dpi. The black asterisks above each data bar indicate the statistical significance of the results: * adjusted *p* < 0.05; ** adjusted *p* < 0.001, Student’s *t*-test (detailed results are presented in Table S2).

The following SAR marker genes were tested: *PAL, NPR1, PR-1b*. A significant decrease in the expression of the *PAL* gene was observed at 5 days after mechanical rubbing of the leaf blade (Figure 4). During the entire experiment, the highest expression increase was demonstrated for the *PR-1b* gene (at 4 hpi and 9 dpi for BTH-, [Chol][BTHCOO]- and BTHWA-treated plants) (Figure 4). Another gene from the PR gene family (*GluB)* that is associated with a pathogen defence response showed increased expression up to 1 day after treatment with each resistance inducer tested. However, after this time, its expression in plants treated with each of the tested resistance inducers began to decrease (Figure 4). Interestingly, in the absence of pathogens, the highest expression of the *GluB* gene was observed after plant treatment with an ionic liquid, [N_111010_][BTHCOO], which showed the weakest SAR induction properties among the tested inducers during the experiment.

**Figure 5:**
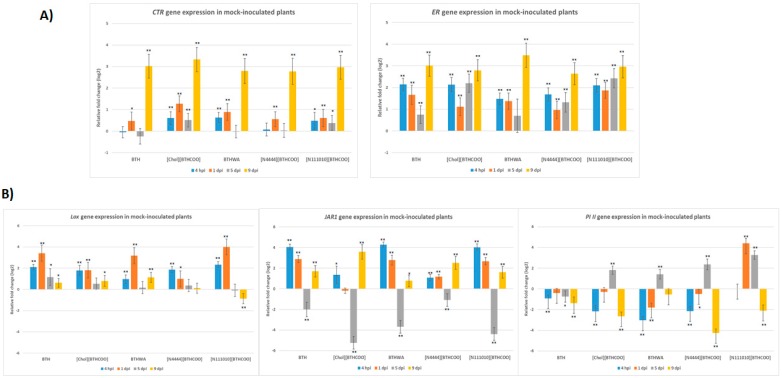
Relative expression changes in: (**A**) two ethylene (ET)-mediated pathway marker genes (*CTR*—serine/threonine-protein kinase and *ER*—ethylene receptor) and (**B**) three JA-mediated pathway marker genes (*LOX*—lipoxygenase, *JAR1*—jasmonic acid-amido synthetase 1 and *PI II—*proteinase inhibitor II) between mock-treated (Plant set 1) and inducer-treated plants (Plant sets 2–6). BTH, benzo(1,2,3)-thiadiazole-7-carbothioic acid S-methyl ester; [Chol][BTHCOO], cholinium benzo(1,2,3)-thiadiazole-7-carboxylate; BTHWA, amide derivative of benzo(1,2,3)-thiadiazole-7-carbothioic acid; [N_4444_][BTHCOO], tetrabutylammonium benzo(1,2,3)-thiadiazole-7-carboxylate; [N_111010_][BTHCOO], didecyldimethylammonium benzo(1,2,3)-thiadiazole-7-carboxylate. Each bar represents differential gene expression on the log2 scale at different time points: 4 h post inoculation (hpi), 1 day post inoculation (dpi), 5 dpi and 9 dpi. The black asterisks above each data bar indicate the statistical significance of the results: * adjusted *p* < 0.05; ** adjusted *p* < 0.001, Student’s *t*-test (results presented in Table S2).

The highest expression levels of marker genes associated with the JA pathway were observed at 4 hpi and 1 dpi, with the exception of the *PI II* gene, which showed an increase in expression at 5 dpi (with the highest expression levels observed in plants treated with two ionic liquids: [N_4444_][BTHCOO] and [N_111010_][BTHCOO]). Low levels of *CTR* gene expression were observed until 5 dpi. In contrast to the *ER* gene, the induction of expression was observed for each analysed day. At 9 dpi, an increase in the levels of expression of both *CTR* and *ER* genes was observed, and this increase was comparable in plants treated with all the tested inducers (Figure 5).

### 2.4. Analysis of the Expression of Defence-Related Marker Genes after BTH and Its Derivatives Treatments in Virus-Infected Plants

Plant leaves (from inoculated to apical) were used to analyse the expression levels of marker genes in the SA-, JA-, and ET-mediated pathways after viral infection (Figure 1, Plant sets 7–12). As shown above in the healthy plants treated with resistance inducers, the expression of these genes in the virus-infected plants was tested and compared to that in water-treated, non-inoculated plants (mock-inoculated control group; Figure 1, Plant set 1). The times presented in the results were calculated from the time of ToMV inoculation, as mentioned above.

**Figure 6:**
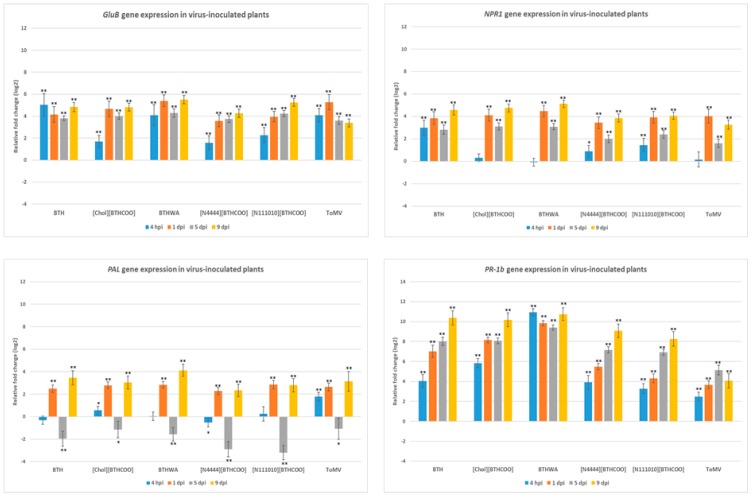
Relative changes in the expression of four salicylic acid (SA)-mediated pathway marker genes *(GluB*—glucan endo-1,3-beta-glucanase; *NPR1*—non-expressor of pathogenesis-related genes 1; *PAL—*phenylalanine ammonia-lyase gene; *PR-1b*—pathogenesis-related 1b) between mock-treated (Plant set 1) and tomato mosaic virus (ToMV)-treated plants (Plant sets 7–12). Tested plants were not treated with any resistance inducer (indicated in figure as ToMV; Plant set 7) or treated with benzo(1,2,3)-thiadiazole-7-carbothioic acid S-methyl ester (BTH); cholinium benzo(1,2,3)-thiadiazole-7-carboxylate ([Chol][BTHCOO]); amide derivative of benzo(1,2,3)-thiadiazole-7-carbothioic acid (BTHWA); tetrabutylammonium benzo(1,2,3)-thiadiazole-7-carboxylate ([N_4444_][BTHCOO]); or didecyldimethylammonium benzo(1,2,3)-thiadiazole-7-carboxylate ([N_111010_][BTHCOO]). Each bar represents differential gene expression on the log2 scale at different time points: 4 h post inoculation (hpi), 1 day post inoculation (dpi), 5 dpi and 9 dpi. The black asterisks above each data bar indicate the statistical significance of the results: * adjusted *p* < 0.05; ** adjusted *p* < 0.001, Student’s *t*-test (results presented in Table S3).

**Figure 7:**
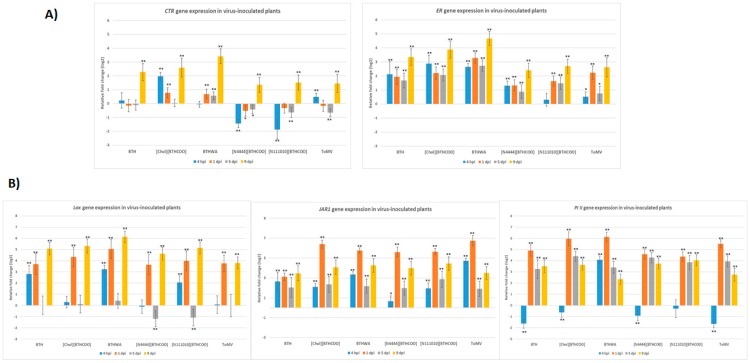
Relative changes in the expression of: (**A**) two ET-mediated pathway marker genes (*CTR*—serine/threonine-protein kinase and *ER—*ethylene receptor) and (**B**) three JA-mediated pathway marker genes (*LOX—*lipoxygenase, *JAR1*—jasmonic acid-amido synthetase 1 and *PI II*—proteinase inhibitor II) between mock-treated (Plant set 1) and tomato mosaic virus (ToMV)-treated plants (Plant sets 7–12). Tested plants were not treated with any resistance inducer (indicated on figure as ToMV; Plant set 7) or treated with benzo(1,2,3)-thiadiazole-7-carbothioic acid S-methyl ester (BTH); cholinium benzo(1,2,3)-thiadiazole-7-carboxylate ([Chol][BTHCOO]); amide derivative of benzo(1,2,3)-thiadiazole-7-carbothioic acid (BTHWA); tetrabutylammonium benzo(1,2,3)-thiadiazole-7-carboxylate ([N_4444_][BTHCOO]); or didecyldimethylammonium benzo(1,2,3)-thiadiazole-7-carboxylate ([N_111010_][BTHCOO]). Each bar represents differential gene expression on the log2 scale at different time points: 4 h post inoculation (hpi), 1 day post inoculation (dpi), 5 dpi and 9 dpi. The black asterisks above each data bar indicate the statistical significance of the results: * adjusted *p* < 0.05; ** adjusted *p* < 0.001, Student’s *t*-test (results presented in Table S3).

The ToMV-infected plants not treated with resistance inducers (Figure 1, Plant set 7) showed the highest expression levels of resistance marker genes at 1 dpi and 9 dpi, except for the *PR-1b* gene, which had an expression level lower than those in all the inducer-treated plants (Figure 6). At 1 dpi and 9 dpi, upregulated expression of the majority of the analysed genes was observed, with the exception of the *CTR* gene, in which an increase in expression was observed only at 9 dpi (Figure 7). The highest increase in gene expression was recorded for the *PR-1b* gene and persisted throughout the duration of the experiment after treatment with all tested resistance inducers, with the highest increase in virus-infected plants treated with BTHWA at all analysed time points after virus inoculation (Figure 6). Additionally, when comparing untreated virus-infected plants to virus-infected plants treated with BTH or its derivatives, a significant increase in *PR-1b* gene expression on each analysed day for all the tested inducers was observed (Figure S1a,b and Table S4). This comparison also showed statistically significant upregulation of the *ER* and *PI II* genes (4 hpi), *CTR, ER* and *NPR1* (5 dpi) and all genes except for the *PAL* gene (9 dpi) in BTHWA- and [Chol][BTHCOO]-treated plants. The analysis also showed statistically significant downregulation of the *JAR1* gene (4 hpi and 1 dpi) and the *PAL* gene (4 hpi). Notably, most of the genes studied (*CTR, ER, LOX, GluB, NPR1* and *PR-1b*) were upregulated at 9 dpi, particularly in BTH-, [Chol][BTHCOO]- and BTHWA-treated plants, which may indicate a continuous induction of resistance signalling in plants and synergistic action of the SA, JA, and ET signalling pathways upon virus and resistance inducer treatment.

To show the influence of resistance inducer treatment on the course of viral infection, a comparative analysis of gene expression in non-infected inducer-treated plants (Figure 1, Plant sets 2–6) and virus-infected inducer-treated plants (Figure 1, Plant sets 8–12) was performed. A significant increase in gene expression was observed from 1 dpi. The virus inoculation caused increased gene expression of *PAL* (1 dpi), *NPR1* (1 dpi, 5 dpi and 9 dpi), *PR-1b* (4 hpi (for BTHWA only), 5 dpi and 9 dpi), *GluB* (5 dpi and 9 dpi), *JAR1* (1 dpi, 5 dpi and 9 dpi), *LOX* (1 dpi and 9 dpi), *PI II* (4 hpi (for BTHWA only), 1 dpi and 9 dpi) and *ER* (mainly for BTHWA at all-time points) and decreases in *PAL* (4 hpi) and *CTR* (1 dpi, 5 dpi and 9 dpi with the exception of BTHWA) gene expression (Figure S2a,b and Table S5), which suggests that these genes are involved in the plant response to ToMV infection.

The results of the analyses of individual resistance marker gene expression responses to viral infection and to the inducer treatment were confirmed using a two-way ANOVA test and are presented in Table S6. Most of the gene expression results for both factors (virus and inducer) were statistically significant at 4 hpi, 1 dpi and 9 dpi. A multivariate ANOVA test was used to confirm the statistical significance of gene expression levels after comparison of three independent variables (viral infection, resistance inducer treatment (BTHWA and [Chol][BTHCOO]), and time point post infection). For both of these BTH derivatives, all results obtained were statistically significant (Table S7).

### 2.5. Viral RNA Accumulation in Plants after Treatment with BTH and Its Derivatives

**Figure 8:**
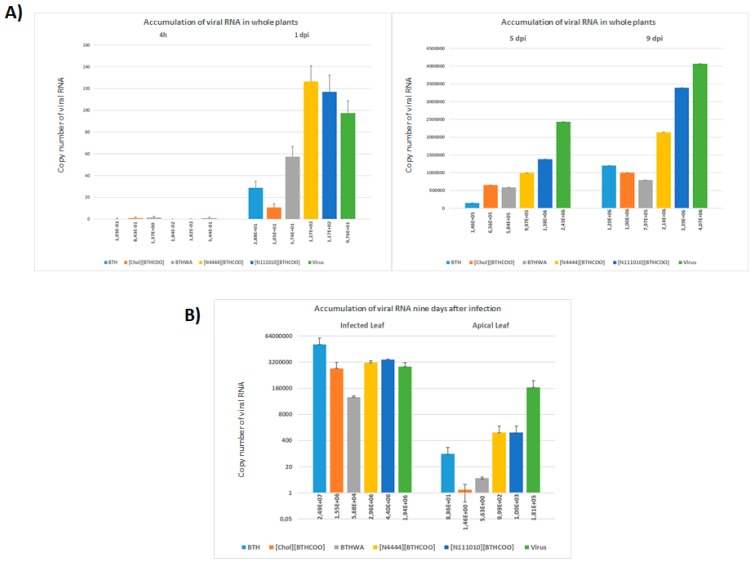
Accumulations of viral RNA in plants treated with the selected resistance inducers; (**A**) accumulation of viral RNA isolated from the whole plant, starting from the inoculated leaf, at every time point of the experiment using the studied resistance inducers; (**B**) accumulation of viral RNA at 9 dpi in the infected leaf and in the apical leaf.

The accumulation of viral RNA in plants treated with water, BTH or its derivatives was analysed. Leaves from the inoculated leaf to the apical leaf were harvested, combined and analysed as a single sample (one sample per inoculated plant treated with water and BTH or its derivatives). In plants treated with the tested resistance inducers, less accumulation of viral RNA was observed at 1 dpi than in the untreated infected plants (Figure 8A), especially for BTH, [Chol][BTHCOO] and BTHWA.

**Figure 9:**
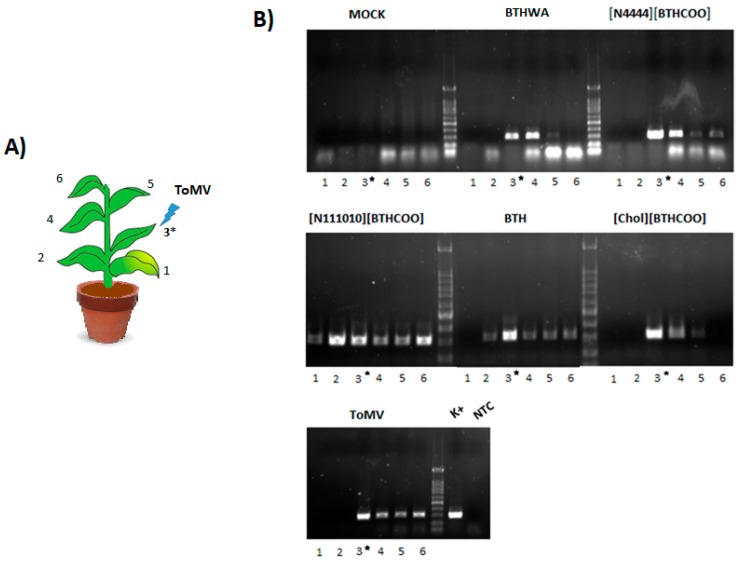
(**A**) Diagram showing the tested plant and inoculated leaf; (**B**) detection of ToMV at 9 dpi in agarose gel (1%) electrophoresis after PCR amplification; lanes 1-6 represent different leaves from a plant; the black asterisk shows the leaf that was inoculated (in negative control plants, it was rubbed with clean water); K+—positive control prepared from virus inoculum, NTC—no template control.

To assess the efficiency of viral RNA transport in plants infected with the virus after challenge with resistance inducers, samples from individual leaves were isolated (from the inoculated leaf to the apical leaf), and the level of viral RNA accumulation was analysed (Figure 8B and Figure 9). Reduced accumulation of viral RNA in the apical leaf was observed for plants challenged with all the resistance inducers tested, with the lowest level of viral RNA accumulation observed for plants pre-treated with BTHWA (approximately 36,000 times less viral RNA in plants challenged with this compound than in water-treated plants with virus inoculation (Figure 1, Plant set 7)) and [Chol][BTHCOO] (approximately 120,000 times less than in water-treated plants). Detection of viral RNA in individual leaves (Figure 9) confirmed a lower accumulation level of viral RNA in plants treated with both these resistance inducers. For the apical leaves of plants pre-treated with BTHWA and [Chol][BTHCOO], the viral RNA was below the level of detection using RT-PCR; therefore, a real-time PCR was performed to confirm a low level of viral RNA accumulation in these leaves.

### 2.6. Effectiveness of Resistance Inducer Application (BTHWA or[Chol][BTHCOO]) to Plants Previously Infected with Virus

**Figure 10:**
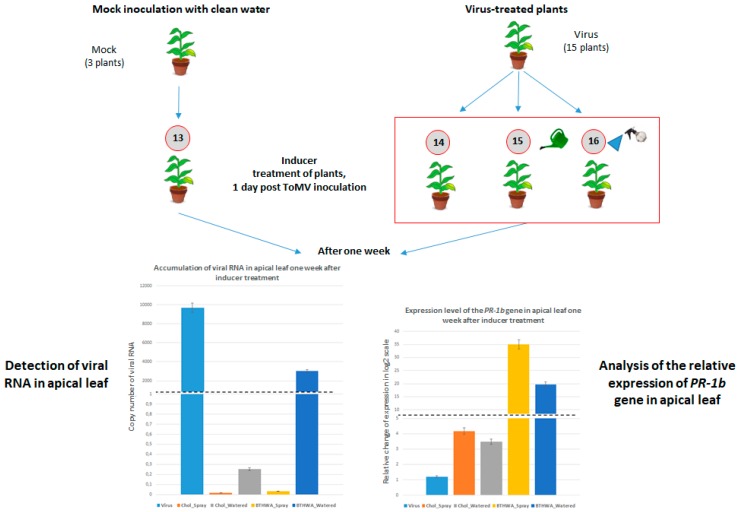
Diagram showing the experimental setup in which virus inoculation predated inducer treatment (BTHWA or[Chol][BTHCOO]); Plant set 13—Mock-inoculated plants not treated with inducer (3 plants), Plant set 14—Virus-inoculated plants not treated with inducer (3 plants), Plant set 15—Virus-inoculated plants watered with BTHWA (3 plants) or [Chol][BTHCOO] (3 plants), Plant set 16—Virus-inoculated plants sprayed with BTHWA (3 plants) or [Chol][BTHCOO] (3 plants). Left diagram: accumulation levels of viral RNA in plants treated with the selected compounds one day after infection by spraying on each leaf or by soil watering using real-time PCR; right diagram: relative expression change in the *PR-1b* gene in *N. tabacum* cv. Samsun inoculated with virus before selected compound treatment by spraying each leaf or by soil watering. Both analyses were performed one week after inducer treatment. The names of the bars from the left: Virus, Chol_Spray (sprayed with [Chol][BTHCOO]), Chol_Watered (watered with [Chol][BTHCOO]), BTHWA_Spray (sprayed with BTHWA), BTHWA_Watered (watered with BTHWA).

In another experiment, plants were first inoculated with ToMV. One day after virus inoculation, BTHWA or [Chol][BTHCOO] was applied to the plants in two different ways (by watering or by spraying on leaves). As a control group, we prepared mock-inoculated plants treated with water (Plant set 13) and virus-inoculated plants treated with water (Plant set 14) (Figure 10). Plants were observed for one week after treatment. Samples were collected from the apical leaves and analysed for the presence and accumulation of viral RNA. A high accumulation level of viral RNA was observed for plant set 14 (virus-inoculated plants treated with water) (Figure 10). When plants were treated with BTHWA or [Chol][BTHCOO] by soil watering, the inducer did not completely stop the systemic transport of the virus; however, their significantly lower level of viral RNA accumulation suggests that viral replication and/or transport was less efficient. Spraying the plants was a more effective way of applying these compounds (Figure 10, left). Additionally, an analysis of *PR-1b* gene expression showed a higher level of expression in plants sprayed with the tested resistance inducers (Figure 10, right).

## 3. Discussion

Modern, intensive agricultural production delivers the majority of the food that the world population consumes. However, to feed all humanity, plants must be protected from harmful pests and pathogens that either feed on plants or cause plant diseases. To limit this danger, pesticides were invented, allowing farmers to fight the organisms that endanger crops. Specific chemical substances can greatly reduce the risk of losses caused by organisms such as insect pests or fungi. By contrast, we are almost helpless against viral infections, as these pathogens are specific in their action and closely associated with host plants; thus, there is no direct method to fight them without harming the plants. Plant viruses are responsible for serious losses in agricultural production worldwide every year [56,57]. The crop damage caused by viruses might reach up to 100% of the yield, as is the case for tomato yellow leaf curl virus (TYLC) infection in tomato fields [58]. These pathogens are intracellular parasites that there is no direct possibility to eliminate. Only preventive approaches might reduce the damage caused by viral infections. From the entry of a pathogen to the spread of disease, little time generally passes, and the symptoms of viral diseases may vary, even between isolates of the same virus [59]. The virus used in our studies, ToMV, can infect a wide range of plant species, including hosts from the Solanaceae (e.g., tomato and pepper), which are closely related to tobacco and are agriculturally important crop species [60]. In the first half of the twentieth century, TMV and ToMV caused significant losses in tomato production [61].

Viral infection causes plant responses that determine their fate. The first publication report of the tobacco plants systemic response to viral infection were made by Ross (1961) [10,24]. Further studies showed that the induction of resistance was non-specific with respect to both the inducing and challenging pathogens [62]. Primary infection of cucumber with the fungus *Colletotrichum lagenarium* or with tobacco necrosis virus (TNV) led to enhanced resistance against fungi, bacteria and viruses causing various foliar and root diseases [63]. Today, a large number of natural [64,65] and chemical [66] resistance inducers have been proposed to stimulate plant defence mechanisms.

In this study, we analysed the influence of BTH and its derivatives on virus-induced pathogenesis and their potential in the activation of plant innate resistance. BTH was used as the base to prepare both ionic liquids, [Chol][BTHCOO], [N_4444_][BTHCOO], and [N_111010_][BTHCOO] [37,38,67,68], and a non-ionic liquid, the amide derivative BTHWA [69]. The changes in the expression levels of resistance marker genes associated with the SA, JA, and ET pathways were studied. During the entire experiment, the symptoms of viral infection developed only on plants infected with the virus and not treated with any studied resistance inducer. Similar results were obtained previously in a local necrotic (hypersensitive reaction) host of TMV, when some of these inducers ([Chol][BTHCOO], [N_4444_][BTHCOO], [N_111010_][BTHCOO]) were tested on *N. tabacum* cv. Xanthi infected with TMV [37,38].

The onset of the induction of plant defence gene expression after pathogen infection is extremely important. A faster response to a pathogenic agent can mitigate infection or even lead to complete recovery of the plant. In our study, a concentration of 10 mg/L was used without any phytotoxic effect and with good SAR induction results. However, too high a dose can lead to a lack of induction of immunity and can have a negative effect on plant fitness, as Kouzai and colleagues showed when they used a BION in a significantly higher dose than ours [70]. Therefore, it should be kept in mind that excessive activation of immunity in plants may shift the delicate metabolic balance to the disadvantage of the plant, weakening it and causing phytotoxic effects. Determining the appropriate proportions and time of dosing for synthetic resistance inducers is very important because their use has many advantages, including a reduction in the use of pesticides [23]. BTH and its derivatives act similarly to SA in plant tissues in the SAR induction process. To analyse their potential to induce SAR, the gene expression of four SAR-related marker genes, *PAL, NPR1, PR-1b* and *GluB*, was studied.

The level of SA production is dependent, among other factors, on the expression of the *PAL* gene [71,72]. *PAL* is a key regulator of the phenylpropanoid pathway (induced under a variety of biotic and abiotic stress conditions) that participates in the production of phenolic compounds with a significant range of biological functions [73]. We observed a low level of expression of the *PAL* gene at 4 hpi and 1 dpi in mock-inoculated plants treated with the tested inducers (Figure 1, Plant sets 2–6), with downregulation in expression at 5 dpi. After ToMV infection, we observed downregulation (or no change) in the expression of the *PAL* gene at 4 hpi and 5 dpi in inducer-treated plants. These results may suggest that the SAR response in inducer-treated plants was *PAL* pathway independent. In contrast, ToMV-infected plants untreated with the studied inducers (Figure 1, Plant set 7) showed higher expression of the *PAL* gene at 4 hpi than plants treated with inducers, which in this case may suggest the participation of this pathway in response to the viral pathogen when inducers were not applied. The high expression level of the *PAL* gene at 9 dpi in mock-inoculated plants treated with inducers and at 1 dpi and 9 dpi in virus-inoculated plants treated with inducers (Figure 1, Plant sets 8–12) may suggest that different strategies were associated with these changes in *PAL* expression [74]. A similar situation was observed by Jin Yu [75]. Namely, in *A. thaliana* treated with the SAR inducer probenazole (PBZ), *PAL* expression was downregulated. However, they observed upregulation in the expression of the isochorismate synthase (*ICS*) gene, which is also involved in the SA synthesis pathway. The authors suggested that PBZ application to *A. thaliana* plants activated the *ICS*-dependent SA synthesis pathway rather than the *PAL*-dependent SA synthesis pathway [75]; however, to confirm this speculation for resistance inducers used in this study, further tests are required.

We also analysed changes in the expression level of the *NPR1* gene, which interacts with the transcription factor TGA in the nucleus, inducing the expression of *PR* genes [76]. SA accumulation in leaves constitutes a signal for the induction of defence-related gene expression to which the family of PR proteins belongs [9]. A slight increase in the level of *NPR1* gene expression and simultaneous high *PR-1b* gene expression was observed in this study. Among the possible explanations for this difference, we can speculate that: (a) the synthesis of the *PR1* gene is induced not only by the increase in *NPR1* gene expression level but also by conformational changes in the NPR1 protein [77]; (b) the gene expression of *NPR1* may have been higher before the first time point chosen for our gene expression analyses; (c) as in *A. thaliana* mutants (*npr1*) treated with BTH, which showed an increase in the expression level of the *PR1* gene, an alternative *PR1* synthesis induction pathway may exist [50]; and (d) *NPR1* may suppress the JA pathway [50,78]. Thus, it is possible that a slight increase in *NPR1* gene expression, as observed in our study, did not cause a decrease in the expression of marker genes in the JA pathway. Low levels of *NPR1* gene expression also suggest the activation of transcription factors involved in the expression of another *PR* gene (such as *PR2*–*GluB*) or other NPR1–independent pathways involved in the plant defence response [79,80]. A lack of noticeable change in the expression level of the *NPR1* gene (in contrast to an increase in the expression level of the *PR-1b* gene) was observed at 4 hpi in ToMV-infected plants not treated with any inducer, which may suggest that viral infection induces *PR-1b* gene expression in *N. tabacum* after the first few hours post infection in an NPR1-independent manner. After 1 dpi, *NPR1*-dependent *PR* expression began in uninduced virus-inoculated plants.

Glucan endo-1,3-beta-glucosidase (*endo-1,3-beta-glucanase; GluB*) is an enzyme that belongs to the PR family, and similar to chitinase, it shows an increase in expression after pathogen infection [80]. This gene is involved in various physiological and developmental processes in plants, including flower formation, fertilization, and seed germination [81]. In addition, *GluB* shows antiviral properties against TMV, probably because of its sequence similarity to TMV-inducible protein [82]. There is evidence that the TMV uses class I β-1,3-glucanase for cell transport [82,83,84]; however, in this work, increased expression of the *GluB* gene was not correlated with increased accumulation of viral RNA in the uninoculated tissues of ToMV-infected plants treated with water or inducers. In contrast, there was a decrease in the accumulation of viral RNA, which may indicate a significant effect of *GluB* gene expression on the course of infection. β-1,3-glucanase is also involved in the deposition of callose in plasmodesmata. By reducing the amount of callose, it allows for greater permeability between cells, which can accelerate the transport of immune signals between the cells and accelerate the plant’s response to the appearance of a pathogen [84,85].

Reports also exist that metabolites involved in the JA synthesis pathway are involved in SAR induction [86]. To initiate a plant defence reaction dependent on JA, the transformation of linoleic acid into oxophytodienoic acid (OPDA) by lipoxygenase occurs, followed by later modifications leading to the formation of JA [50]. After JA synthesis, the *JAR1* and *cytochrome c oxidase I (COI)* genes are expressed. *JAR1* modifies JA into JA-Ile, which interacts with the jasmonate ZIM domain (*JAZ)* and activates transcription factors (MYC2, 3, 4) [87,88]. These transcription factors in turn activate the expression of the *plant defensin 1.2 (PDF1.2)* gene, *PIN-FORMED (PIN1)* gene or *PI II* gene [50]. The high expression of the *LOX* and *JAR1* genes at 1 dpi and 9 dpi and the *PI II* gene from 1 dpi to 9 dpi after virus appearance may suggest the involvement of the JA pathway in response to ToMV. It was postulated previously that TMV infection on tobacco plants induces proteinase inhibitor gene expression, which is involved in HR response and formation of TMV-induced local lesions [89]. One very interesting and important phenomenon is the increased induction of *PI II* gene expression after virus inoculation compared to mock-inoculated plants, which may indicate the effect of this gene in the response of tobacco plants to ToMV infection. The high expression level at 9 dpi of JA-related marker genes after virus inoculation may suggest a positive influence of the tested inducers on the activation of various types of plant defence response to the pathogen and indicate a longer duration of action.

Ethylene, which is involved in many processes related to plant biology, such as seed germination, seedling growth, organ development and senescence, leaf and petal abscission, fruit ripening, stress and pathogen responses [90], interacts closely with JA. Ethylene also plays a role as a signalling molecule in the first stages of response to damage or pathogens. The accumulation of *CTR1* and *ER* gene transcripts activates *ethylene-insensitive protein 2 (EIN2)* or *ethylene response (ETR)* gene expression, which in turn initiates the synthesis of the *PDF1.2* gene (positive crosstalk between the JA and ET pathways) [50]. *ER* interacts with ethylene, which activates the kinase cascade reaction, in which, among others, the *CTR1* gene is involved. An increase in the expression of the *ER* gene suggests an accumulation of ethylene in plant tissue upon mechanical damage to the leaf blade and viral penetration into plant cells, which activates signalling, probably through a *CTR1*-independent pathway (because of the low expression level of the *CTR1* gene before 9 dpi). The high expression of the *CTR1* gene at 9 dpi may indicate the activation of an additional pathway (*CTR1*-dependent) of response to ToMV. This variety of changes suggests that the studied resistance inducers are multifunctional and activate plant defence responses at different levels.

In general, the crosstalk between signalling pathways (SA and JA/ET) is considered to be complex [42,43,44]. It was previously suggested that alpha-momorcharin (α-MMC) treatment before TMV infection causes positive crosstalk between the SA signalling pathway and the JA signalling pathway, which stimulates the plant’s response to viral infection [91]. The α-MMC is a type-I ribosome inactivating protein (29 kDa) that was found in *Momordica charantia*. It was tested in the *M. charantia*–cucumber mosaic virus pathosystem. The crosstalk between SA and JA depends on many factors (including the type of pathogen, environmental factors, and plant condition) [40,92,93]. Microarray analyses of defence responses in *Arabidopsis thaliana* showed correlations among the biosynthetic pathways of SA, JA and ET, which were not necessarily antagonistic [43]. Plants infected with ToMV after treatment with BTHWA, [Chol][BTHCOO], [N_4444_][BTHCOO] and [N_111010_][BTHCOO] did not show inhibition of JA biosynthesis (increased expression level of *JAR1, LOX and PI II* genes was observed). SAR induction and activation of the expression of ISR-related genes in plants treated with the tested inducers may allow for synergistic crosstalk between the SA- and JA-related pathways [94]. The increase in the expression level of the majority of the tested genes at 9 dpi (when we compared virus-infected plants treated with water to virus-infected plants treated with inducers (Figure S1)) also confirmed the hypothesis of positive crosstalk between SA and JA. An increase in *PI II* gene expression was found to be associated, among other factors, with insect feeding on plant tissues [95]. In this study, increased *PI II* expression was also observed in infected plants treated with each resistance inducer tested, which may influence plant-insect interactions [96]. Additionally, when we compared the expression level of the *PI II* gene in virus-inoculated plants treated with inducers to that in virus-inoculated plants treated with water, we observed the largest difference for BTHWA after 4 hpi, which suggests that amide derivatives of BTH have the greatest potential to induce resistance against feeding pests. This potential needs to be tested further.

No defence mechanism appears to operate against virus entry; it is considered passive [97]. This is why early SAR induction in plants is so important. We found that all of the tested inducers caused SAR induction according to the expression of defence marker genes before virus inoculation, but none were able to completely stop virus transport. However, all of them greatly reduced the level of ToMV RNA accumulation in leaves (Figure 8). In the majority of published studies, and in our study, using resistance inducers lead to a gradual reduction of the replication rate of the pathogen [86,98,99], which may lead to the later recovery of the plant [100]. Potential new SAR activators should have three main characteristics: (a) the substance induces resistance against all pathogens encountered in the environment; (b) neither the compound nor its metabolites should have antimicrobial activity; and (c) chemical treatment should induce expression of the same biochemical markers as in the biological model [101]. Interestingly, ionic liquids, due to their structure, are considered less stable compounds. However, in our case, [Chol][BTHCOO], which is an ionic liquid, turned out to be one of the most effective inducers of resistance, together with the amide derivative of BTH (BTHWA). The effect of SAR induction in plants treated with these inducers was observed for one month without any symptoms of disease after viral infection. Additionally, the effects of BTHWA and [Chol][BTHCOO] induction on plant defence marker gene expression lasted the longest, which could also be observed in the reduced level of virus accumulation in plants treated with these inducers. These two compounds even showed good efficacy when applied to plants the day after virus inoculation, which suggests the possibility of application of resistance inducers after contact with a virus to protect the rest of a crop against damage resulting from a spreading infection.

In this study, we have shown the multifunctionality of plant SAR inducers derived from the BTH molecule. The best-performing inducers among these BTH derivatives were BTHWA and [Chol][BTHCOO]. Through activation of the expression of various defence genes associated with different signalling pathways (SA, JA, ET) and reduced viral RNA accumulation, we have demonstrated the positive impact of the tested compounds on induced resistance in plants. Activation of these signalling pathways could decrease the threat of virus expansion and development and increase the chance of limiting the damage caused by a broad spectrum of pathogens and pests. This potential, however, needs to be confirmed in further studies. We demonstrated that the appropriate dosing method may limit viral infection, which may lead to host plant recovery. Many different factors might affect the effectiveness of inducers, including: (a) selection of the optimal inducer concentration; (b) appropriate modification of the inducer, which will increase its properties without disturbing its structure; (c) plant fitness and developmental stage, because damaged and weakened plants do not always endure the treatment; (d) plant type; (e) time and method of dosing; and (f) number of pathogens found in the environment. The use of inducers lessens the burden on the environment and can provide a good substitute for the use of pesticides as plant protection products in the future.

### 4.1. BTH and Its Derivatives

The following resistance inducers were chosen to test BTH [38] and its derivatives: (a) three ionic liquids, cholinium benzo(1,2,3)-thiadiazole-7-carboxylate ([Chol][BTHCOO]) [38,67], tetrabutylammonium benzo(1,2,3)-thiadiazole-7-carboxylate ([N_4444_][BTHCOO]), didecyldimethylammonium benzo(1,2,3)-thiadiazole-7-carboxylate ([N_111010_][BTHCOO]) [37,68] and (b) amide derivative of benzo(1,2,3)-thiadiazole-7-carbothioic acid (information on this substance is proprietary (soon to be published) [69]). All chemical compounds mentioned were prepared at Poznań Science and Technology Park, Adam Mickiewicz University Foundation (Poland). To investigate phytotoxic effects on plants, four concentrations of BTH and its derivatives were tested: 5, 10, 25, 50 mg/L, and applied to the soil by watering (100 mL). The optimal concentration of compounds was determined to be 10 mg/L and was used in further experiments by watering (100 mL).

### 4.2. Plants and Virus Materials

In this study, the experimental model consisted of a host plant (*Nicotiana tabacum* cv. Samsun), a plant virus (ToMV), and the resistance inducer (BTH or its derivatives). Seedlings of tobacco plants were maintained in greenhouse conditions in the Research Centre of Quarantine, Invasive and Genetically Modified Organisms in the Institute of Plant Protection—National Research Institute in Poznań. Plants were grown at 26 °C with a 12-h day/ 12-h night cycle.

In all experiments, the SL-1 isolate of ToMV was used. To obtain a viral inoculum, *N. tabacum* (cv. Samsun) plants were sap-inoculated with previously virus-infected plant material. After the appearance of disease symptoms on the infected plants (leaf mosaic), the virus was isolated by sucrose density gradient centrifugation with 0.01 M Tris-HCl, pH 7.0 [102] to prepare the infectious inoculum of the virus. The concentration of virus in the obtained preparation was determined spectrophotometrically. The samples of ToMV were stored at −20 °C until used.

### 4.3. BTH and Its Derivatives Treatments of Plants Followed by Subsequent Virus Inoculation

Six- to eight-week-old plants at the three-mature-leaf stage were used in the experiments. For each tested variant, thirty-two plants were used (four biological replicates for each sampling point: four hours, one day, five days and nine days post inoculation; 16 plants for each plant set; Figure 1). One week after the plants were watered once with solution containing the tested resistance inducer at a final concentration of 10 mg/L or with water (negative control), the plants were evenly dusted with carborundum followed by: (a) rubbing with clean water (Figure 1, Plant sets 1–6) or (b) virus inoculation (Figure 1, Plant sets 7–12). Plants were inoculated with the ToMV inoculum at a concentration of 7.2 × 10^−4^ ng per leaf. Samples from all leaves (including inoculated and apical leaves) were harvested at each time point, frozen in liquid nitrogen and stored at −80 °C until use. The experiment was repeated three times.

### 4.4. BTH and Its Derivatives Treatments of Plants Following Prior Virus Inoculation

*N. tabacum* plants previously infected with ToMV were watered once (100 mL) or sprayed once with 10 mg/L BTHWA and [Chol][BTHCOO] one day after inoculation (three biological replicates for each experimental variant; Figure 10). One week after virus inoculation, apical leaf samples were harvested and tested for the presence of viral RNA and *PR-1b* gene expression. The experiments were performed three times for each tested inducer.

### 4.5. RNA Isolation and cDNA Synthesis

Leaf samples were collected samples and ground, and RNA was isolated using Tri Reagent (Life Technologies) as described previously [103]. The isolated RNA was suspended in 50 µL of RNase-free water. Total RNA concentration was estimated using a NanoDrop ND-1000 (Thermo Scientific, Waltham, MA, USA) spectrophotometer.

The extract was digested in the presence of 5 U of RNase-free DNase I (Thermo Scientific), and the purified RNA (2 µg) was used for cDNA synthesis using the RevertAid RT Reverse Transcription Kit (Thermo Scientific). One microlitre of RNA was added to 100 µM random hexamers (Thermo Scientific), and then the solution was heat-denatured at 65 °C for 5 min and rapidly cooled on ice. The samples were spun quickly, and reverse transcription mix containing 5 × buffer (250 mM Tris-HCl (pH 8.3), 250 mM KCl, 20 mM MgCl_2_, 50 mM DTT), 2 µL of 10 mM dNTPs, 20 U of RiboLock RNase Inhibitor and 200 U of RevertAid Reverse Transcriptase (Thermo Scientific) was added. The samples were incubated first at 25 °C for 10 min, then at 42 °C for 60 min, and finally, the reaction was terminated at 75 °C for 5 min. The obtained cDNA samples were diluted with equal volumes of water and used for real-time PCR.

### 4.6. Analysis of SA, JA, and ET Pathway-Related Gene Expression

The relative expression of four SA pathway marker genes, *PAL*, *NPR1*, *GluB* and *PR-1b*; three JA pathway marker genes, *LOX*, *JAR1* and *PI II*; and two ET pathway marker genes, *CTR* and *ER*, were analysed in water- and inducer (BTH and its derivatives)-treated plants, as well as in plants treated both with resistance inducers and ToMV. The gene expression of an *N. tabacum* housekeeping gene, elongation factor-1 alpha (*EF-1α*), was used for validation. Real-time PCR was performed with gene-specific primers designed by using the Primer3 (v. 0.4.0) software [104] (Table S1). Reactions were performed in a final volume of 10 µL of a master mix containing 2× iTaq™ Universal SYBR® Green Supermix (Bio-Rad, CA, USA), 400 nM forward and reverse primers and 1 µL of cDNA obtained in the previous step. PCR thermal cycling was performed as follows: 1 min of initial denaturation at 95 °C followed by 40 cycles of 10 s at 95 °C, 20 s annealing at the temperatures given in Table S1 and 15 s at 72 °C. Melting curves were generated during a temperature ramp from 65 to 95 °C. Ct data were collected automatically by the software supplied with the LightCycler^®^ 480 Real-Time PCR System (Roche, Basel, Switzerland). Relative expression changes in analysed genes were calculated using ΔΔ*C*_T_ methods in the Rest 2009 Software (Qiagen, Hilden, Germany) and in the GenEx version 6 (Multid Analyses AB, Göteborg, Sweden) program.

### 4.7. Analysis of Viral RNA Accumulation

Samples were taken from the inoculated plants 4 h and 1, 5 and 9 days after infection. RNA was isolated for cDNA synthesis as described before. The accumulation of viral RNA was assessed using a real-time PCR approach as described above with primers designed for a fragment of the helicase gene of ToMV (Table S1). The reaction was normalized using the standard curve method, and the Ct data received after real-time PCR were recalculated in Microsoft Excel to obtain the absolute expression of viral RNA.

### 4.8. Statistical Analysis

Gene expression studies were performed for four biological replicates, and each was performed in three technical replicates. The Ct data were exported to a table matrix in Microsoft Excel and used for the subsequent analyses. An independent Student’s *t*-test was conducted to test for significant differences between inducer (BTH and its derivatives)- and water-treated samples in all experiments. One-way ANOVA was conducted to test for significant differences between the mock-inoculated plants and the plants treated with each inducer. Two-way ANOVA and multivariate ANOVA tests were conducted to test for significant differences in gene expression levels with respect to the analysis of two independent variables: virus inoculation and inducer treatment. ANOVA tests were performed using the STATISTICA program (StatSoft). The *P*-value was calculated for each experiment. The experiment was repeated three times.

## Figures and Tables

**Table 1:** Phytotoxicity of tested resistance inducers.

Compound/Concentration	5 mg/L	10 mg/L	25 mg/L	50 mg/L
BTH	-	-	*	+
[Chol][BTHCOO]	-	-	*	+
BTHWA	-	-	*	+
[N_4444_][BTHCOO]	-	-	*	+
[N_111010_][BTHCOO]	-	-	*	+

“-“, no phytotoxicity observed; “*”, low phytotoxicity after three weeks of observation; “+”, strong phytotoxicity after one week (tissue damage observed).

## Supplementary Materials

Supplementary materials can be found at https://www.mdpi.com/1422-0067/20/7/1598/s1.

## Abbreviations

BTH	benzo(1,2,3)-thiadiazole-7-carbothioic acid S-methyl ester
SAR	systemic acquired resistance
SA	salicylic acid
PAL	phenylalanine ammonia-lyase
NPR1	non-expressor of PR genes
PR	pathogenesis-related
JA	jasmonic acid
ET	ethylene
LAR	localized acquired resistance
ISR	induced systemic resistance
TMV	Tobacco mosaic virus
PGPR	plant growth-promoting rhizobacteria
MeSA	methyl salicylic acid
AzA	azelaic acid
DA	dihydroabetinal
G3P	glycerol-3-phosphate
PIP	pipecolic acid
GluB	glucan endo-1,3-beta-glucanase
TGA	basic leucine zipper transcription factor
ER	ethylene receptor
CTR	serine/threonine protein kinase
JAR1	jasmonic acid-amido synthetase 1
LOX	lipoxygenase
PI II	proteinase inhibitor II
HR	hypersensitive response
PCD	programmed cell death
OPDA	oxophytodienoic acid
ToMV	Tomato mosaic virus
MP	movement protein
CP	coat protein
JAZ	jasmonate ZIM domain
[Chol][BTHCOO]	liquid cholinium benzo(1,2,3)-thiadiazole-7-carboxylate
[N_4444_][BTHCOO]	tetrabutylammonium benzo(1,2,3)-thiadiazole-7-carboxylate
[N_111010_][BTHCOO]	didecyldimethylammonium benzo(1,2,3)-thiadiazole-7-carboxylate
BTHWA	amide derivative of benzo(1,2,3)-thiadiazole-7-carbothioic acid
α-MMC	alpha-momorcharin
ASM	acibenzolar-*S*-methyl
SABP2	salicylic acid binding protein 2
PBZ	probenazole
ICS	isochorismate synthase
