# Supplementary material for: Assessment of the Efficacy and Mode of Action of Benzo(1,2,3)-Thiadiazole-7-Carbothioic Acid S-Methyl Ester (BTH) and Its Derivatives in Plant Protection Against Viral Disease
Source: Int J Mol Sci. 2019 Mar 30;20(7):1598. doi: 10.3390/ijms20071598 (PMC6480033; doi:10.3390/ijms20071598)
Supplement: Supplementary file 1 [file ijms-20-01598-s001.pdf]

Supplementary Materials

Assessment of the efficacy and mode of action of benzo(1,2,3)-thiadiazole-7-carbiothioic acid S-methyl ester (BTH) and its derivatives in plant protection against viral diseases

Patryk Frąckowiak[1,] Henryk Pospieszny[2], Marcin Śmiglak[3], Aleksandra Obręplaska-Stęplowska[1*]

1. Department of Entomology, Animal Pests and Biotechnology, Institute of Plant Protection – National Research Institute, Poznań, Poland
2. Department of Virology and Bacteriology, Institute of Plant Protection – National Research Institute, Poznań, Poland
3. Poznań Science and Technology Park, Adam Mickiewicz University Foundation, ul. Rubież 46 61-612 Poznań, Poland

* Correspondence to: Aleksandra Obręplaska-Stęplowska, e-mail: olaob@o2.pl

Table S1. Primers used in the study.

Analyzed genes	Gene	Primer pair	Sequence (5'->3')	Size (bp)	% of primers efficiency	Tm	Access number
ET pathway marker genes	*CTR*	Forward	TAACGATCCCAGCTTGAAGG	161	110	58°C	Nicotiana tabacum CTR mRNA, partial cds; **GenBank: AF247567.1**
		Reverse	CCTTCGACATGGAAACCAAG				
	ER	Forward	ACGAAGCGCTATGCTTTGAT	182	100	64°C	Nicotiana tabacum ethylene receptor (LOC107784797), mRNA; **NCBI Reference Sequence: NM_001325388.1**
		Reverse	AGAGCGACATTCTGCTCCAT				
JA pathway marker genes	*Lox*	Forward	CTCATCAGTGCTGACCATGC	150	97	53°C	Nicotiana tabacum LOX mRNA for lipoxygenase; **GenBank: AB233415.1**
		Reverse	CATGGACTCATCCCAGTCAA				
	JAR	Forward	GGCTTGCATTTTTCAAAGGA	166	120	53°C	Nicotiana tabacum jasmonic acid-amido synthetase JAR1-like (LOC107762553), mRNA; **NCBI Reference Sequence: XM_016580920.1**
		Reverse	ATACCGCTTTTCGCATTTTG				
	PI II	Forward	CACGGGATACAAAGGTTGCT	180	100	56°C	N.tabacum (Samsun NN) gene for proteinase inhibitor II; **GenBank: Z29537.1**
		Reverse	CAACCCTTGTCTGCGTTACA				
SA pathway marker genes	*gluB*	Forward	GATCAGGAGGGGCATCTGTA	248	99	60°C	Nicotiana tabacum glucan endo-1,3-beta-glucosidase, basic vacuolar (LOC107823411), mRNA; **NCBI Reference Sequence: XM_016650053.1**
		Reverse	ATATTTGGGCTGCTTGTTGG				
	NPR1	Forward	GTAGGCCAGCTGTAGCGTTC	175	98	64°C	Nicotiana tabacum NPR1 mRNA, complete cds; **GenBank: KY402167.1**
		Reverse	CTCTCGCATGCTTTACCACA				
	PAL	Forward	AGTCGTGGACAGGGAATACG	141	105	64°C	phenylalanine ammonia-lyase (LOC104230518), mRNA; **NCBI Reference Sequence: XM_009783351.1**
		Reverse	ATTGAGCTGTTCGCGTTCTT				
	PR-1b	Forward	GCAGACTGCAACCTCGTACA	213	97	58°C	pathogenesis-related protein 1b (PR1b); **NCBI Reference Sequence: X03465.1**
		Reverse	CCTAGCACATCCAACACGAA				
Reference Gene	*EF-1α*	Forward	TGTGATGTTTTTGTTCGGTCTTTTAA	180	100	60°C	elongation factor 1-alpha (LOC107826390), mRNA; **NCBI Reference Sequence: NM_001326165.1**
		Reverse	TCAAAAGAAAATGCAGACAGACTCA				
Virus polimerase	*Hel_ToMV*	Forward	ATTATCTGGCGACGTTCCTG	239	99	63°C	Tomato mosaic virus complete genome (helicase fragment); **NCBI Reference Sequence: FN985165.1**
		Reverse	CAAGGTCAATCGCAGCTGTA				

Table S2. Statistical analysis of relative defence marker gene expression in *N. tabacum* cv. Samsun not infected with virus after BTH/derivative treatment (4 hours, 1 day, 5 days, and 9 days) between mock-treated and inducer-treated plants. A) SA marker genes, B) ET marker genes, C) JA marker genes. BTH, benzo(1,2,3)-thiadiazole-7-carbothioic acid S-methyl ester; [Chol][BTHCOO], cholinium benzo(1,2,3)-thiadiazole-7-carboxylate; BTHWA, amide derivative of benzo(1,2,3)-thiadiazole-7-carbothioic acid; [N_{4444}][BTHCOO], tetrabutylammonium benzo(1,2,3)-thiadiazole-7-carboxylate; [N_{111010}][BTHCOO], didecyldimethylammonium benzo(1,2,3)-thiadiazole-7-carboxylate. The different colours in each column indicate the statistical significance of the results: red – no statistical significance; yellow - adjusted *P<0.05;* green - adjusted *P<0.001*, Student's t-test.

A)

(BTH) vs (Mock)	gluB 4h	gluB 1dpi	gluB 5dpi	gluB 9dpi	NPR1 4h	NPR1 1dpi	NPR1 5dpi	NPR1 9dpi	PAL 4h	PAL 1dpi	PAL 5dpi	PAL 9dpi	PR-1b 4h	PR-1b 1dpi	PR-1b 5dpi	PR-1b 9dpi
P-Value	1.00E-08	1.00E-08	1.00E-08	0.0359899	1.00E-08	1.00E-08	1.00E-08	1.00E-08	1.00E-08	1.00E-08	0.0016644	1.00E-08	1.00E-08	1.00E-08	1.00E-08	1.00E-08
([Chol][BTHCOO]) vs (Mock)	gluB 4h	gluB 1dpi	gluB 5dpi	gluB 9dpi	NPR1 4h	NPR1 1dpi	NPR1 5dpi	NPR1 9dpi	PAL 4h	PAL 1dpi	PAL 5dpi	PAL 9dpi	PR-1b 4h	PR-1b 1dpi	PR-1b 5dpi	PR-1b 9dpi
P-Value	1.00E-08	1.30E-06	1.00E-08	2.70E-08	1.00E-08	0.0003951	1.00E-08	1.00E-08	1.00E-08	0.1968133	0.0001881	1.00E-08	1.00E-08	1.00E-08	1.00E-08	1.00E-08
(BTHWA) vs (Mock)	gluB 4h	gluB 1dpi	gluB 5dpi	gluB 9dpi	NPR1 4h	NPR1 1dpi	NPR1 5dpi	NPR1 9dpi	PAL 4h	PAL 1dpi	PAL 5dpi	PAL 9dpi	PR-1b 4h	PR-1b 1dpi	PR-1b 5dpi	PR-1b 9dpi
P-Value	8.70E-08	1.00E-08	3.50E-05	0.1872596	0.0092349	1.00E-08	1.00E-08	1.00E-08	4.94E-05	1.00E-08	2.31E-05	1.00E-08	1.00E-08	1.00E-08	1.00E-08	1.00E-08
([N4444][BTHCOO]) vs (Mock)	gluB 4h	gluB 1dpi	gluB 5dpi	gluB 9dpi	NPR1 4h	NPR1 1dpi	NPR1 5dpi	NPR1 9dpi	PAL 4h	PAL 1dpi	PAL 5dpi	PAL 9dpi	PR-1b 4h	PR-1b 1dpi	PR-1b 5dpi	PR-1b 9dpi
P-Value	1.00E-08	1.00E-08	1.00E-08	1.00E-08	1.00E-08	6.13E-05	4.10E-08	0.0053226	5.57E-07	0.1157627	8.33E-05	1.00E-08	1.00E-08	1.00E-08	1.00E-08	1.00E-08
([N111010][BTHCOO]) vs (Mock)	gluB 4h	gluB 1dpi	gluB 5dpi	gluB 9dpi	NPR1 4h	NPR1 1dpi	NPR1 5dpi	NPR1 9dpi	PAL 4h	PAL 1dpi	PAL 5dpi	PAL 9dpi	PR-1b 4h	PR-1b 1dpi	PR-1b 5dpi	PR-1b 9dpi
P-Value	1.00E-08	1.00E-08	1.00E-08	9.08E-06	1.00E-08	1.00E-08	1.00E-08	1.00E-08	4.74E-06	1.00E-08	0.0073994	0.0012852	1.00E-08	1.00E-08	1.00E-08	1.00E-08

B)

(BTH) vs (Mock)	CTR 4h	CTR 1dpi	CTR 5dpi	CTR 9dpi	ER 4h	ER 1dpi	ER 5dpi	ER 9dpi
P-Value	0.37554	0.0331548	0.1276676	1.00E-08	1.00E-08	5.00E-08	0.0015814	1.00E-08
([Chol][BTHCOO]) vs (Mock)	CTR 4h	CTR 1dpi	CTR 5dpi	CTR 9dpi	ER 4h	ER 1dpi	ER 5dpi	ER 9dpi
P-Value	0.0005796	3.08E-07	0.0045109	1.00E-08	1.00E-08	1.68E-05	1.00E-08	1.00E-08
(BTHWA) vs (Mock)	CTR 4h	CTR 1dpi	CTR 5dpi	CTR 9dpi	ER 4h	ER 1dpi	ER 5dpi	ER 9dpi
P-Value	2.46E-05	0.0001772	0.4586663	1.00E-08	1.00E-08	7.80E-08	0.0678142	1.00E-08
([N4444][BTHCOO]) vs (Mock)	CTR 4h	CTR 1dpi	CTR 5dpi	CTR 9dpi	ER 4h	ER 1dpi	ER 5dpi	ER 9dpi
P-Value	0.335228	0.0044137	0.4246884	1.00E-08	1.00E-08	0.0001146	4.92E-06	1.00E-08
([N111010][BTHCOO]) vs (Mock)	CTR 4h	CTR 1dpi	CTR 5dpi	CTR 9dpi	ER 4h	ER 1dpi	ER 5dpi	ER 9dpi
P-Value	0.019647	0.0060547	0.037889	1.00E-08	1.00E-08	1.00E-08	1.00E-08	1.00E-08

C)

(BTH) vs (Mock)	Lox 4h	Lox 1dpi	Lox 5dpi	Lox 9dpi	JAR1 4h	JAR1 1dpi	JAR1 5dpi	JAR1 9dpi	PI II 4h	PI II 1dpi	PI II 5dpi	PII 9dpi
P-Value	1.00E-08	1.00E-08	0.0187257	0.0169013	1.00E-08	1.00E-08	1.38E-05	3.72E-06	6.62E-06	0.1332618	0.0276048	0.0003499
([Chol][BTHCOO]) vs (Mock)	Lox 4h	Lox 1dpi	Lox 5dpi	Lox 9dpi	JAR1 4h	JAR1 1dpi	JAR1 5dpi	JAR1 9dpi	PI II 4h	PI II 1dpi	PI II 5dpi	PII 9dpi
P-Value	4.80E-08	8.82E-05	0.0545426	0.0062947	0.0046936	0.1159092	1.00E-08	1.00E-08	5.60E-08	0.1133836	1.00E-08	1.00E-08
(BTHWA) vs (Mock)	Lox 4h	Lox 1dpi	Lox 5dpi	Lox 9dpi	JAR1 4h	JAR1 1dpi	JAR1 5dpi	JAR1 9dpi	PI II 4h	PI II 1dpi	PI II 5dpi	PII 9dpi
P-Value	6.48E-05	1.00E-08	0.3146388	0.0001202	1.00E-08	1.00E-08	1.00E-08	0.0137954	1.00E-08	1.00E-08	1.70E-06	0.0568919
([N4444][BTHCOO]) vs (Mock)	Lox 4h	Lox 1dpi	Lox 5dpi	Lox 9dpi	JAR1 4h	JAR1 1dpi	JAR1 5dpi	JAR1 9dpi	PI II 4h	PI II 1dpi	PI II 5dpi	PII 9dpi
P-Value	1.00E-08	0.0147883	0.1434205	0.3376273	8.90E-08	1.00E-08	0.0022699	1.20E-08	1.00E-08	0.0370724	1.00E-08	1.00E-08
([N111010][BTHCOO]) vs (Mock)	Lox 4h	Lox 1dpi	Lox 5dpi	Lox 9dpi	JAR1 4h	JAR1 1dpi	JAR1 5dpi	JAR1 9dpi	PI II 4h	PI II 1dpi	PI II 5dpi	PII 9dpi
P-Value	1.00E-08	1.00E-08	0.3953637	0.0020904	1.00E-08	1.00E-08	1.00E-08	9.95E-06	0.4815915	1.00E-08	1.00E-08	2.00E-08

Table S3. Statistical analysis of relative defence marker gene expression in *N. tabacum* cv. Samsun infected with virus after BTH/derivative treatment (4 hours, 1 day, 5 days, and 9 days) between mock-treated and virus-inoculated inducer-treated plants. A) SA marker genes, B) ET marker genes, C) JA marker genes. ToMV, Virus-inoculated plants not treated with any resistance inducer; BTH, benzo(1,2,3)-thiadiazole-7-carbothioic acid S-methyl ester; [Chol][BTHCOO], cholinium benzo(1,2,3)-thiadiazole-7-carboxylate; BTHWA, amide derivative of benzo(1,2,3)-thiadiazole-7-carbothioic acid; [N_{4444}][BTHCOO], tetrabutylammonium benzo(1,2,3)-thiadiazole-7-carboxylate; [N_{111010}][BTHCOO], didecyldimethylammonium benzo(1,2,3)-thiadiazole-7-carboxylate. The different colours in each column indicate the statistical significance of the results: red – no statistical significance; yellow - adjusted *P<0.05;* green - adjusted *P<0.001*, Student's t-test.

A)

(BTH) vs (Mock)	gluB 4h	gluB 1dpi	gluB 5dpi	gluB 9dpi	NPR1 4h	NPR1 1dpi	NPR1 5dpi	NPR1 9dpi	PAL 4h	PAL 1dpi	PAL 5dpi	PAL 9dpi	PR-1b 4h	PR-1b 1dpi	PR-1b 5dpi	PR-1b 9dpi
P-Value	1.00E-08	1.00E-08	1.00E-08	1.00E-08	1.00E-08	1.00E-08	1.00E-08	1.00E-08	0.072458	1.00E-08	5.57E-06	1.00E-08	1.00E-08	1.00E-08	1.00E-08	1.00E-08
([Chol][BTHCOO]) vs (Mock)	gluB 4h	gluB 1dpi	gluB 5dpi	gluB 9dpi	NPR1 4h	NPR1 1dpi	NPR1 5dpi	NPR1 9dpi	PAL 4h	PAL 1dpi	PAL 5dpi	PAL 9dpi	PR-1b 4h	PR-1b 1dpi	PR-1b 5dpi	PR-1b 9dpi
P-Value	2.18E-06	1.00E-08	1.00E-08	1.00E-08	0.076592	1.00E-08	1.00E-08	1.00E-08	0.00433	1.00E-08	0.005145	1.00E-08	1.00E-08	1.00E-08	1.00E-08	1.00E-08
(BTHWA) vs (Mock)	gluB 4h	gluB 1dpi	gluB 5dpi	gluB 9dpi	NPR1 4h	NPR1 1dpi	NPR1 5dpi	NPR1 9dpi	PAL 4h	PAL 1dpi	PAL 5dpi	PAL 9dpi	PR-1b 4h	PR-1b 1dpi	PR-1b 5dpi	PR-1b 9dpi
P-Value	1.00E-08	1.00E-08	1.00E-08	1.00E-08	0.324528	1.00E-08	1.00E-08	1.00E-08	0.434208	1.00E-08	3.42E-05	1.00E-08	1.00E-08	1.00E-08	1.00E-08	1.00E-08
([N4444][BTHCOO]) vs (Mock)	gluB 4h	gluB 1dpi	gluB 5dpi	gluB 9dpi	NPR1 4h	NPR1 1dpi	NPR1 5dpi	NPR1 9dpi	PAL 4h	PAL 1dpi	PAL 5dpi	PAL 9dpi	PR-1b 4h	PR-1b 1dpi	PR-1b 5dpi	PR-1b 9dpi
P-Value	7.06E-05	1.00E-08	1.00E-08	1.00E-08	0.003469	1.00E-08	1.00E-08	1.00E-08	0.01325	1.00E-08	1.00E-08	1.00E-08	1.00E-08	1.00E-08	1.00E-08	1.00E-08
([N111010][BTHCOO]) vs (Mock)	gluB 4h	gluB 1dpi	gluB 5dpi	gluB 9dpi	NPR1 4h	NPR1 1dpi	NPR1 5dpi	NPR1 9dpi	PAL 4h	PAL 1dpi	PAL 5dpi	PAL 9dpi	PR-1b 4h	PR-1b 1dpi	PR-1b 5dpi	PR-1b 9dpi
P-Value	8.72E-07	1.00E-08	1.00E-08	1.00E-08	8.00E-05	1.00E-08	1.00E-08	1.00E-08	0.259323	1.00E-08	1.00E-08	1.00E-08	1.00E-08	1.00E-08	1.00E-08	1.00E-08
(ToMV) vs (Mock)	gluB 4h	gluB 1dpi	gluB 5dpi	gluB 9dpi	NPR1 4h	NPR1 1dpi	NPR1 5dpi	NPR1 9dpi	PAL 4h	PAL 1dpi	PAL 5dpi	PAL 9dpi	PR-1b 4h	PR-1b 1dpi	PR-1b 5dpi	PR-1b 9dpi
P-Value	1.00E-08	1.00E-08	1.00E-08	1.00E-08	0.350232	1.00E-08	1.00E-08	1.00E-08	1.00E-08	1.00E-08	0.03165	1.42E-07	1.00E-08	1.00E-08	1.00E-08	1.00E-08

B)

(BTH) vs (Mock)	CTR 4h	CTR 1dpi	CTR 5dpi	CTR 9dpi	ER 4h	ER 1dpi	ER 5dpi	ER 9dpi
P-Value	0.250416	0.29479	0.318387	6.30E-08	0.000121	1.07E-07	1.48E-06	1.00E-08
([Chol][BTHCOO]) vs (Mock)	CTR 4h	CTR 1dpi	CTR 5dpi	CTR 9dpi	ER 4h	ER 1dpi	ER 5dpi	ER 9dpi
P-Value	1.00E-08	0.0003	0.383719	3.20E-08	1.00E-08	1.00E-08	1.00E-08	1.00E-08
(BTHWA) vs (Mock)	CTR 4h	CTR 1dpi	CTR 5dpi	CTR 9dpi	ER 4h	ER 1dpi	ER 5dpi	ER 9dpi
P-Value	0.342048	0.000902	0.000946	1.00E-08	1.00E-08	1.00E-08	1.00E-08	1.00E-08
([N4444][BTHCOO]) vs (Mock)	CTR 4h	CTR 1dpi	CTR 5dpi	CTR 5dpi	ER 4h	ER 1dpi	ER 5dpi	ER 9dpi
P-Value	1.00E-08	0.014658	0.013865	0.013865	1.50E-08	4.82E-07	0.00167	1.00E-08
([N111010][BTHCOO]) vs (Mock)	CTR 4h	CTR 1dpi	CTR 5dpi	CTR 9dpi	ER 4h	ER 1dpi	ER 5dpi	ER 9dpi
P-Value	2.12E-05	0.06655	0.002224	1.27E-05	0.133079	1.00E-08	5.52E-07	1.00E-08
(ToMV) vs (Mock)	CTR 4h	CTR 1dpi	CTR 5dpi	CTR 9dpi	ER 4h	ER 1dpi	ER 5dpi	ER 9dpi
P-Value	0.001466	0.253346	0.000169	0.000306	0.00792	1.00E-08	0.007656	3.20E-08

C)

(BTH) vs (Mock)	Lox 4h	Lox 1dpi	Lox 5dpi	Lox 9dpi	JAR1 4h	JAR1 1dpi	JAR1 5dpi	JAR1 9dpi	PI II 4h	PI II 1dpi	PI II 5dpi	PII 9dpi
P-Value	1.00E-08	1.00E-08	0.480441	1.00E-08	1.14E-07	1.00E-08	9.00E-05	1.00E-08	1.00E-08	1.00E-08	1.00E-08	1.00E-08
([Chol][BTHCOO]) vs (Mock)	Lox 4h	Lox 1dpi	Lox 5dpi	Lox 9dpi	JAR1 4h	JAR1 1dpi	JAR1 5dpi	JAR1 9dpi	PI II 4h	PI II 1dpi	PI II 5dpi	PII 9dpi
P-Value	0.128496	1.00E-08	0.378128	1.00E-08	1.00E-08	1.00E-08	1.94E-07	1.00E-08	0.003113	1.00E-08	1.00E-08	1.00E-08
(BTHWA) vs (Mock)	Lox 4h	Lox 1dpi	Lox 5dpi	Lox 9dpi	JAR1 4h	JAR1 1dpi	JAR1 5dpi	JAR1 9dpi	PI II 4h	PI II 1dpi	PI II 5dpi	PII 9dpi
P-Value	1.00E-08	1.00E-08	0.104201	1.00E-08	1.00E-08	1.00E-08	8.60E-08	1.00E-08	1.00E-08	1.00E-08	1.00E-08	1.00E-08
([N4444][BTHCOO]) vs (Mock)	Lox 4h	Lox 1dpi	Lox 5dpi	Lox 9dpi	JAR1 4h	JAR1 1dpi	JAR1 5dpi	JAR1 9dpi	PI II 4h	PI II 1dpi	PI II 5dpi	PII 9dpi
P-Value	0.376801	1.00E-08	0.00067	1.00E-08	0.008957	1.00E-08	4.11E-07	1.00E-08	8.08E-05	1.00E-08	1.00E-08	1.00E-08
([N111010][BTHCOO]) vs (Mock)	Lox 4h	Lox 1dpi	Lox 5dpi	Lox 9dpi	JAR1 4h	JAR1 1dpi	JAR1 5dpi	JAR1 9dpi	PI II 4h	PI II 1dpi	PI II 5dpi	PII 9dpi
P-Value	1.00E-08	1.00E-08	0.002342	1.00E-08	1.00E-08	1.00E-08	1.00E-08	1.00E-08	0.23641	1.00E-08	1.00E-08	1.00E-08
(ToMV) vs (Mock)	Lox 4h	Lox 1dpi	Lox 5dpi	Lox 9dpi	JAR1 4h	JAR1 1dpi	JAR1 5dpi	JAR1 9dpi	PI II 4h	PI II 1dpi	PI II 5dpi	PII 9dpi
P-Value	0.423363	1.00E-08	0.490149	1.00E-08	1.00E-08	1.00E-08	4.69E-05	1.00E-08	1.00E-08	1.00E-08	1.00E-08	1.00E-08

.

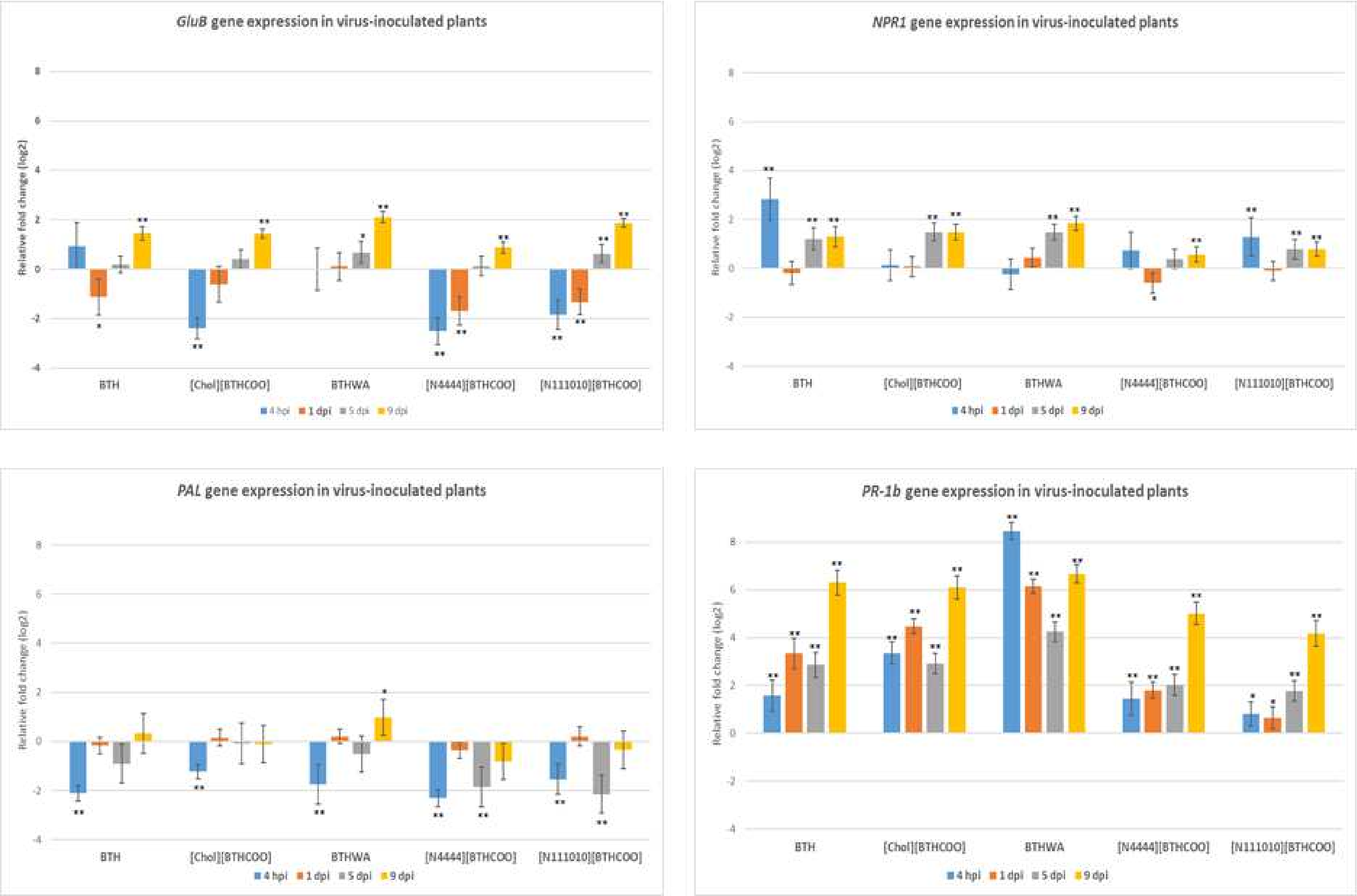

Figure S1a. Relative expression changes in four SA marker genes (GluB - glucan endo-1,3-beta-glucanase; NPR1 – non-expressor of pathogenic-related genes 1; PAL - phenylalanine ammonia-lyase gene; PR-1b – pathogenesis-related 1b) between virus-inoculated water-treated (Plant set 7) and virus-inoculated inducer-treated plants (Plant sets 8-12). BTH, benzo(1,2,3)-thiadiazole-7-carbothioic acid S-methyl ester; [Chol][BTHCOO], cholinium benzo(1,2,3)-thiadiazole-7-carboxylate; BTHWA, amide derivative of benzo(1,2,3)-thiadiazole-7-carbothioic acid; [N4444][BTHCOO], tetrabutylammonium benzo(1,2,3)-thiadiazole-7-carboxylate; [N111010][BTHCOO], didecyldimethylammonium benzo(1,2,3)-thiadiazole-7-carboxylate. Each bar represents differential gene expression on the log2 scale at different time points: 4 hours post inoculation (hpi), 1 day post inoculation (dpi), 5 dpi and 9 dpi. The black asterisks above each data bar indicate the statistical significance of the results: * - adjusted P<0.05; ** - adjusted P<0.001, Student's t-test (detailed results presented in Table S4).

A)

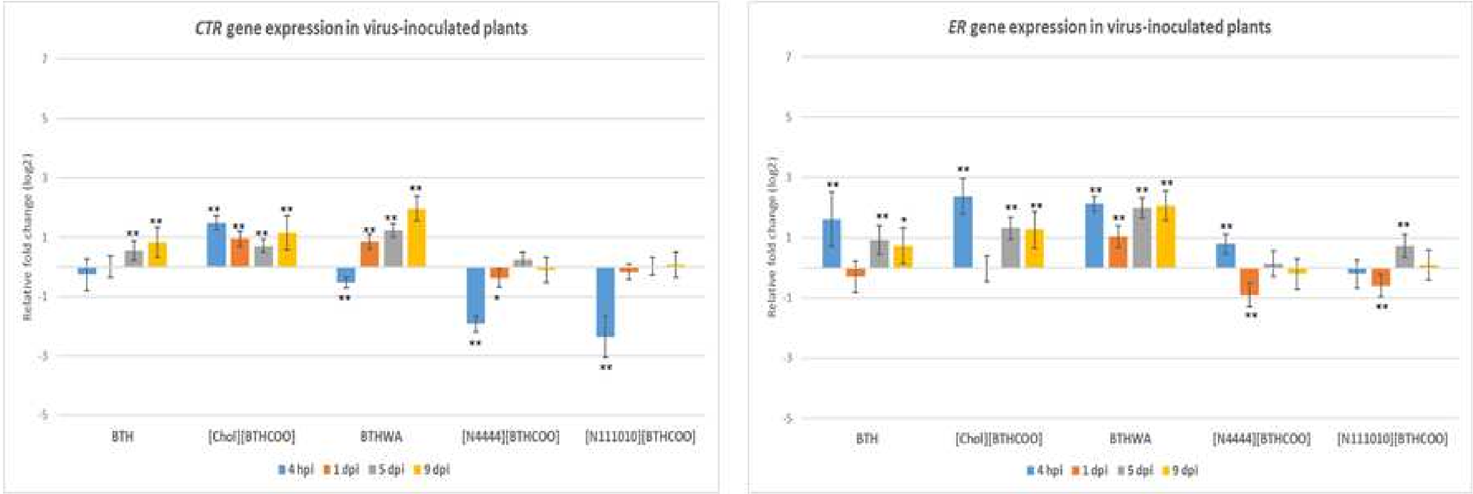

B)

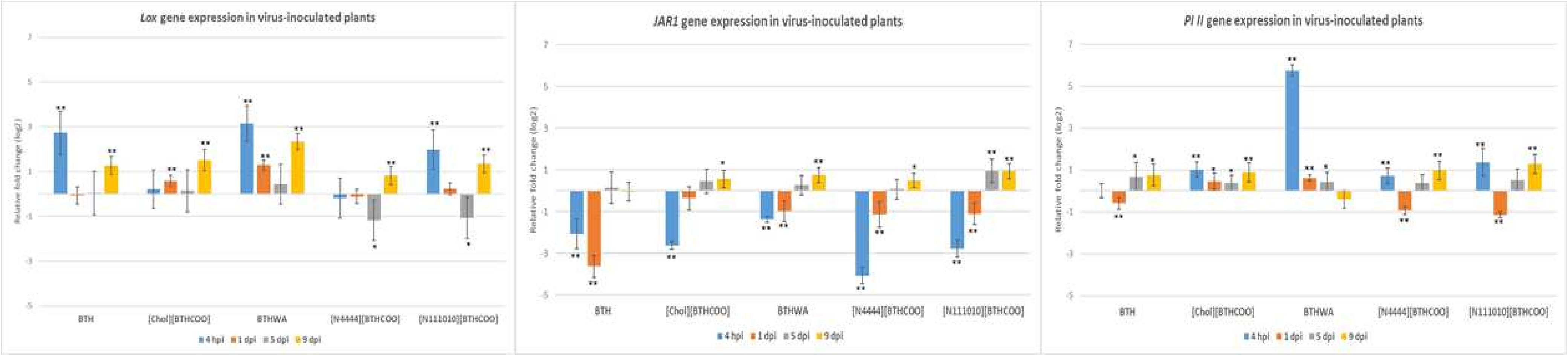

Figure S1b. Relative changes in the expression of two ET-mediated pathway marker genes (CTR - serine/threonine-protein kinase and ER – ethylene receptor) and three JA-mediated pathway marker genes (LOX – lipoxygenase, JAR1 - jasmonic acid-amido synthetase 1 and PI II – proteinase inhibitor II) between virus-inoculated water-treated (Plant set 7) and virus-inoculated inducer-treated plants (Plant sets 8-12). BTH, benzo(1,2,3)-thiadiazole-7-carbothioic acid S-methyl ester; [Chol][BTHCOO], cholinium benzo(1,2,3)-thiadiazole-7-carboxylate; BTHWA, amide derivative of benzo(1,2,3)-thiadiazole-7-carbothioic acid; [N4444][BTHCOO], tetrabutylammonium benzo(1,2,3)-thiadiazole-7-carboxylate; [N111010][BTHCOO] didecyldimethylammonium benzo(1,2,3)-thiadiazole-7-carboxylate. Each bar represents differential gene expression on the log2 scale at different time points: 4 hours post inoculation (hpi), 1 day post inoculation (dpi), 5 dpi and 9 dpi. The black asterisks above each data bar indicate the statistical significance of the results: * - adjusted P<0.05; ** - adjusted P<0.001, Student's t-test (detailed results presented in Table S4).

Table S4. Statistical analysis of relative defence marker gene expression between virus-inoculated water-treated (Plant set 7) and virus-inoculated inducer-treated plants (Plant sets 8-12). A) SA marker genes, B) ET marker genes, C) JA marker genes. BTH, benzo(1,2,3)-thiadiazole-7-carbothioic acid S-methyl ester; [Chol][BTHCOO], cholinium benzo(1,2,3)-thiadiazole-7-carboxylate; BTHWA, amide derivative of benzo(1,2,3)-thiadiazole-7-carbothioic acid; [N4444][BTHCOO], tetrabutylammonium benzo(1,2,3)-thiadiazole-7-carboxylate; ([N111010][BTHCOO], didecyldimethylammonium benzo(1,2,3)-thiadiazole-7-carboxylate. The different colours in each column indicate the statistical significance of the results: red – no statistical significance; yellow - adjusted P<0.05; green - adjusted P<0.001, Student's t-test.

A)

(BTH) vs (ToMV)	gluB 4h	gluB 1dpi	gluB 5dpi	gluB 9dpi	NPR1 4h	NPR1 1dpi	NPR1 5dpi	NPR1 9dpi	PAL 4h	PAL 1dpi	PAL 5dpi	PAL 9dpi	PR-1b 4h	PR-1b 1dpi	PR-1b 5dpi	PR-1b 9dpi
P-Value	0.1022133	0.0129721	0.3094889	1.00E-08	1.21E-06	0.5225064	3.94E-05	3.94E-06	1.00E-08	0.4382324	0.0627319	0.5022811	0.0002145	1.00E-08	1.00E-08	1.00E-08
([Chol][BTHCOO]) vs (ToMV)	gluB 4h	gluB 1dpi	gluB 5dpi	gluB 9dpi	NPR1 4h	NPR1 1dpi	NPR1 5dpi	NPR1 9dpi	PAL 4h	PAL 1dpi	PAL 5dpi	PAL 9dpi	PR-1b 4h	PR-1b 1dpi	PR-1b 5dpi	PR-1b 9dpi
P-Value	1.00E-08	0.1633249	0.0848851	1.00E-08	0.7022308	0.7497585	1.00E-08	1.00E-08	1.00E-08	0.4259199	0.8689251	0.8288641	1.00E-08	1.00E-08	1.00E-08	1.00E-08
(BTHWA) vs (ToMV)	gluB 4h	gluB 1dpi	gluB 5dpi	gluB 9dpi	NPR1 4h	NPR1 1dpi	NPR1 5dpi	NPR1 9dpi	PAL 4h	PAL 1dpi	PAL 5dpi	PAL 9dpi	PR-1b 4h	PR-1b 1dpi	PR-1b 5dpi	PR-1b 9dpi
P-Value	> 0.99	0.7287516	0.0145975	1.00E-08	0.5087756	0.059742	1.00E-08	1.00E-08	1.00E-08	0.2502995	0.2568153	0.0298824	1.00E-08	1.00E-08	1.00E-08	1.00E-08
([N4444][BTHCOO]) vs (ToMV)	gluB 4h	gluB 1dpi	gluB 5dpi	gluB 9dpi	NPR1 4h	NPR1 1dpi	NPR1 5dpi	NPR1 9dpi	PAL 4h	PAL 1dpi	PAL 5dpi	PAL 9dpi	PR-1b 4h	PR-1b 1dpi	PR-1b 5dpi	PR-1b 9dpi
P-Value	1.00E-08	9.78E-06	0.5778086	4.10E-08	0.1039919	0.0179814	0.1066846	0.0046858	1.00E-08	0.0692995	0.0003637	0.0731448	0.0011508	1.00E-08	1.00E-08	1.00E-08
([N111010][BTHCOO]) vs (ToMV)	gluB 4h	gluB 1dpi	gluB 5dpi	gluB 9dpi	NPR1 4h	NPR1 1dpi	NPR1 5dpi	NPR1 9dpi	PAL 4h	PAL 1dpi	PAL 5dpi	PAL 9dpi	PR-1b 4h	PR-1b 1dpi	PR-1b 5dpi	PR-1b 9dpi
P-Value	5.63E-06	7.32E-05	0.0065546	1.00E-08	0.0084321	0.6743439	0.001541	3.86E-05	0.0001205	0.3623759	2.78E-05	0.468785	0.0107152	0.0222585	1.00E-08	1.00E-08

B)

(BTH) vs (ToMV)	CTR 4h	CTR 1dpi	CTR 5dpi	CTR 9dpi	ER 4h	ER 1dpi	ER 5dpi	ER 9dpi
P-Value	0.2124906	0.4741122	0.0028642	0.0044578	0.0020944	0.1810046	0.001023	0.0214926
([Chol][BTHCOO]) vs (ToMV)	CTR 4h	CTR 1dpi	CTR 5dpi	CTR 9dpi	ER 4h	ER 1dpi	ER 5dpi	ER 9dpi
P-Value	1.00E-08	9.00E-08	5.27E-07	0.0009391	1.60E-08	0.4617897	1.72E-07	0.0005973
(BTHWA) vs (ToMV)	CTR 4h	CTR 1dpi	CTR 5dpi	CTR 9dpi	ER 4h	ER 1dpi	ER 5dpi	ER 9dpi
P-Value	3.09E-06	3.61E-07	1.00E-08	1.00E-08	1.00E-08	7.56E-06	1.00E-08	1.00E-08
([N4444][BTHCOO]) vs (ToMV)	CTR 4h	CTR 1dpi	CTR 5dpi	CTR 9dpi	ER 4h	ER 1dpi	ER 5dpi	ER 9dpi
P-Value	1.00E-08	0.0236981	0.0601838	0.3330938	0.0001029	0.0001579	0.2941013	0.2474376
([N111010][BTHCOO]) vs (ToMV)	CTR 4h	CTR 1dpi	CTR 5dpi	CTR 9dpi	ER 4h	ER 1dpi	ER 5dpi	ER 9dpi
P-Value	3.03E-07	0.1380989	0.442908	0.3963237	0.2291278	0.0041317	0.0010968	0.3961425

C)

(BTH) vs (ToMV)	Lox 4h	Lox 1dpi	Lox 5dpi	Lox 9dpi	JAR1 4h	JAR1 1dpi	JAR1 5dpi	JAR1 9dpi	PI II 4h	PI II 1dpi	PI II 5dpi	PII 9dpi
P-Value	1.03E-05	0.3854832	0.4760331	1.71E-06	6.37E-06	1.00E-08	0.3802822	0.4374465	0.4397371	0.0004158	0.0489507	0.0088884
([Chol][BTHCOO]) vs (ToMV)	Lox 4h	Lox 1dpi	Lox 5dpi	Lox 9dpi	JAR1 4h	JAR1 1dpi	JAR1 5dpi	JAR1 9dpi	PI II 4h	PI II 1dpi	PI II 5dpi	PII 9dpi
P-Value	0.3400275	0.0002309	0.4056405	1.20E-06	1.00E-08	0.1482719	0.0935386	0.0172181	7.19E-06	0.0328613	0.0258249	0.0009325
(BTHWA) vs (ToMV)	Lox 4h	Lox 1dpi	Lox 5dpi	Lox 9dpi	JAR1 4h	JAR1 1dpi	JAR1 5dpi	JAR1 9dpi	PI II 4h	PI II 1dpi	PI II 5dpi	PII 9dpi
P-Value	1.50E-08	1.00E-08	0.2064759	1.00E-08	1.00E-08	0.0007724	0.1820283	0.0005518	1.00E-08	1.00E-08	0.021882	0.0649566
([N4444][BTHCOO]) vs (ToMV)	Lox 4h	Lox 1dpi	Lox 5dpi	Lox 9dpi	JAR1 4h	JAR1 1dpi	JAR1 5dpi	JAR1 9dpi	PI II 4h	PI II 1dpi	PI II 5dpi	PII 9dpi
P-Value	0.3648553	0.2684675	0.0170907	0.0007782	1.00E-08	0.0013834	0.3976812	0.0119618	0.0009386	1.00E-08	0.0860447	0.0002577
([N111010][BTHCOO]) vs (ToMV)	Lox 4h	Lox 1dpi	Lox 5dpi	Lox 9dpi	JAR1 4h	JAR1 1dpi	JAR1 5dpi	JAR1 9dpi	PI II 4h	PI II 1dpi	PI II 5dpi	PII 9dpi
P-Value	0.0002439	0.0832981	0.0288582	3.24E-07	1.00E-08	0.0004212	0.0039398	3.82E-05	0.0005039	1.00E-08	0.3844498	9.73E-06

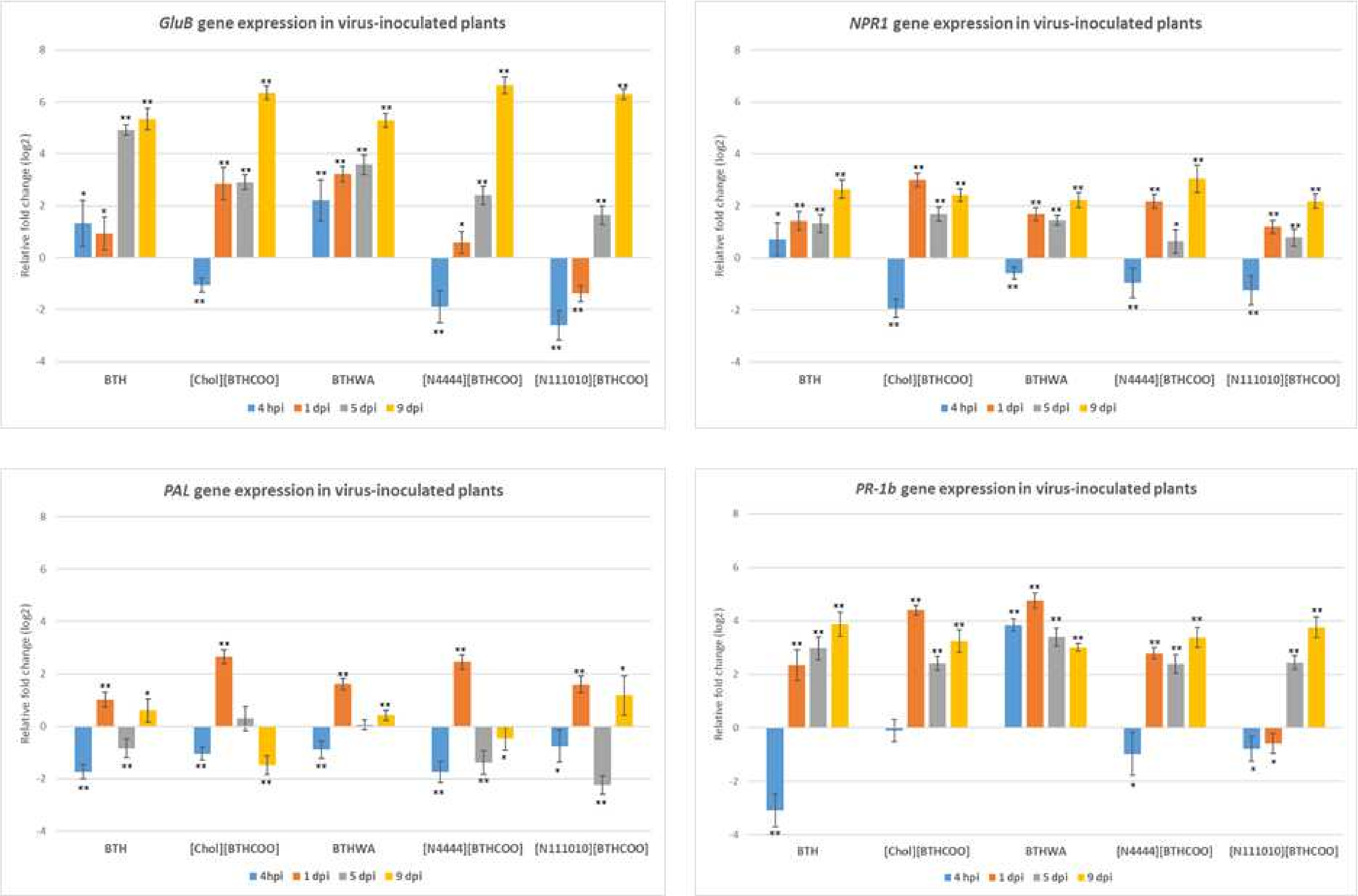

Figure S2a. Relative expression changes in four SA marker genes (GluB - glucan endo-1,3-beta-glucanase; NPR1 – non-expressor of pathogenic-related genes 1; PAL - phenylalanine ammonia-lyase gene; PR-1b – pathogenesis-related 1b) between mock-inoculated inducer-treated (Plant sets 2-6) and virus-inoculated inducer-treated plants (Plant sets 8-12). BTH, benzo(1,2,3)-thiadiazole-7-carbothioic acid S-methyl ester; [Chol][BTHCOO], cholinium benzo(1,2,3)-thiadiazole-7-carboxylate; BTHWA, amide derivative of benzo(1,2,3)-thiadiazole-7-carbothioic acid; [N4444][BTHCOO], tetrabutylammonium benzo(1,2,3)-thiadiazole-7-carboxylate; [N111010][BTHCOO], didecyldimethylammonium benzo(1,2,3)-thiadiazole-7-carboxylate. Each bar represents differential gene expression on the log2 scale at different time points: 4 hours post inoculation (hpi), 1 day post inoculation (dpi), 5 dpi and 9 dpi. The black asterisks above each data bar indicate the statistical significance of the results: * - adjusted P<0.05; ** - adjusted P<0.001, Student's t-test (detailed results presented in Table S5).

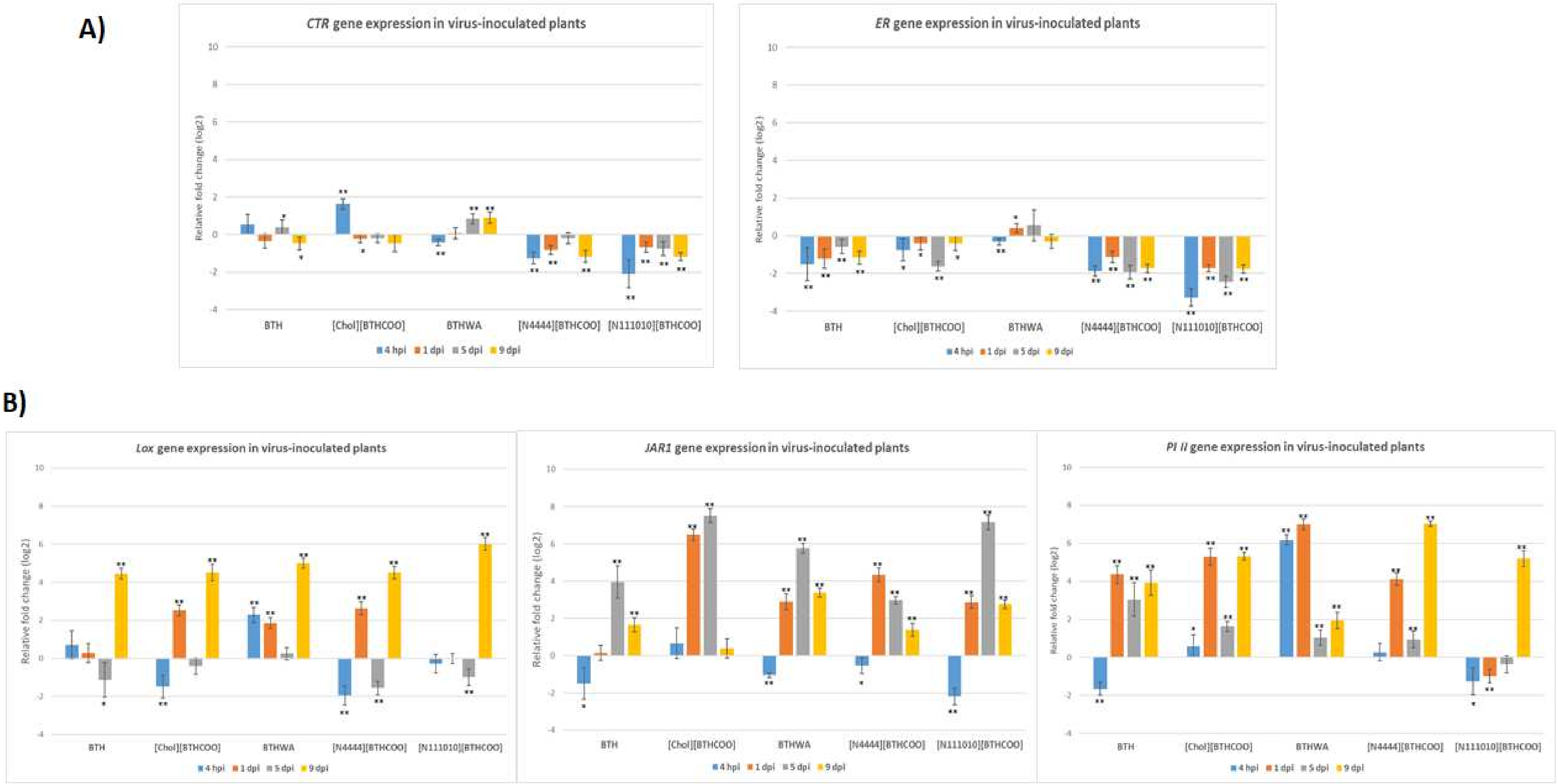

Figure S2b. Relative expression changes in two ET-mediated pathway marker genes (CTR - serine/threonine-protein kinase and ER – ethylene receptor) and three JA-mediated pathway marker genes (LOX – lipoxygenase, JAR1 - jasmonic acid-amido synthetase 1 and PI II – proteinase inhibitor II) between mock-inoculated inducer-treated (Plant sets 2-6) and virus-inoculated inducer-treated plants (Plant sets 8-12). BTH, benzo(1,2,3)-thiadiazole-7-carbothioic acid S-methyl ester; [Chol][BTHCOO], cholinium benzo(1,2,3)-thiadiazole-7-carboxylate; BTHWA, amide derivative of benzo(1,2,3)-thiadiazole-7-carbothioic acid; [N4444][BTHCOO], tetrabutylammonium benzo(1,2,3)-thiadiazole-7-carboxylate; [N111010][BTHCOO], didecyldimethylammonium benzo(1,2,3)-thiadiazole-7-carboxylate. Each bar represents differential gene expression on the log2 scale at different time points: 4 hours post inoculation (hpi), 1 day post inoculation (dpi), 5 dpi and 9 dpi. The black asterisks above each data bar indicate the statistical significance of the results: * - adjusted P<0.05; ** - adjusted P<0.001, Student's t-test (detailed results presented in Table S5).

Table S5. Statistical analysis of relative defence marker gene expression between mock-inoculated inducer-treated (Plant sets 2-6) and virus-inoculated inducer-treated plants (Plant sets 8-12). A) SA marker genes, B) ET marker genes, C) JA marker genes. BTH, benzo(1,2,3)-thiadiazole-7-carbothioic acid S-methyl ester; [Chol][BTHCOO], cholinium benzo(1,2,3)-thiadiazole-7-carboxylate; BTHWA, amide derivative of benzo(1,2,3)-thiadiazole-7-carbothioic acid; [N4444][BTHCOO], tetrabutylammonium benzo(1,2,3)-thiadiazole-7-carboxylate; [N111010][BTHCOO], didecyldimethylammonium benzo(1,2,3)-thiadiazole-7-carboxylate. The different colours in each column indicate the statistical significance of the results: red – no statistical significance; yellow - adjusted $P<0.05$; green - adjusted $P<0.001$, Student's t-test.

A)

(BTH_ToMV) vs (BTH)	gluB 4h	gluB 1dpi	gluB 5dpi	gluB 9dpi	NPR1 4h	NPR1 1dpi	NPR1 5dpi	NPR1 9dpi	PAL 4h	PAL 1dpi	PAL 5dpi	PAL 9dpi	PR-1b 4h	PR-1b 1dpi	PR-1b 5dpi	PR-1b 9dpi
P-Value	0.0081954	0.0087642	1.00E-08	1.00E-08	0.027583	2.10E-08	3.50E-08	1.00E-08	1.00E-08	1.26E-07	0.0001702	0.0127686	1.00E-08	1.00E-08	1.00E-08	1.00E-08
([Chol][BTHCOO]_ToMV) vs ([Chol][BTHCOO])	gluB 4h	gluB 1dpi	gluB 5dpi	gluB 9dpi	NPR1 4h	NPR1 1dpi	NPR1 5dpi	NPR1 9dpi	PAL 4h	PAL 1dpi	PAL 5dpi	PAL 9dpi	PR-1b 4h	PR-1b 1dpi	PR-1b 5dpi	PR-1b 9dpi
P-Value	1.30E-08	1.00E-08	1.00E-08	1.00E-08	1.00E-08	1.00E-08	1.00E-08	1.00E-08	1.00E-08	1.00E-08	0.1494929	1.00E-08	0.3441713	1.00E-08	1.00E-08	1.00E-08
(BTHWA_ToMV) vs (BTHWA)	gluB 4h	gluB 1dpi	gluB 5dpi	gluB 9dpi	NPR1 4h	NPR1 1dpi	NPR1 5dpi	NPR1 9dpi	PAL 4h	PAL 1dpi	PAL 5dpi	PAL 9dpi	PR-1b 4h	PR-1b 1dpi	PR-1b 5dpi	PR-1b 9dpi
P-Value	1.28E-05	1.00E-08	1.00E-08	1.00E-08	5.04E-05	1.00E-08	1.00E-08	1.00E-08	1.74E-05	1.00E-08	0.2914447	0.000343	1.00E-08	1.00E-08	1.00E-08	1.00E-08
([N4444][BTHCOO]_ToMV) vs ([N4444][BTHCOO])	gluB 4h	gluB 1dpi	gluB 5dpi	gluB 9dpi	NPR1 4h	NPR1 1dpi	NPR1 5dpi	NPR1 9dpi	PAL 4h	PAL 1dpi	PAL 5dpi	PAL 9dpi	PR-1b 4h	PR-1b 1dpi	PR-1b 5dpi	PR-1b 9dpi
P-Value	9.70E-08	0.0034058	1.00E-08	1.00E-08	0.0006542	1.00E-08	0.0033715	1.00E-08	1.00E-08	1.00E-08	1.11E-07	0.021929	0.00896	1.00E-08	1.00E-08	1.00E-08
([N111010][BTHCOO]_ToMV) vs ([N111010][BTHCOO])	gluB 4h	gluB 1dpi	gluB 5dpi	gluB 9dpi	NPR1 4h	NPR1 1dpi	NPR1 5dpi	NPR1 9dpi	PAL 4h	PAL 1dpi	PAL 5dpi	PAL 9dpi	PR-1b 4h	PR-1b 1dpi	PR-1b 5dpi	PR-1b 9dpi
P-Value	1.00E-08	1.00E-08	1.00E-08	1.00E-08	0.0002755	1.00E-08	9.67E-05	1.00E-08	0.0192094	1.00E-08	1.00E-08	0.0060288	0.0038279	0.0065431	1.00E-08	1.00E-08

B)

(BTH_ToMV) vs (BTH)	CTR 4h	CTR 1dpi	CTR 5dpi	CTR 9dpi	ER 4h	ER 1dpi	ER 5dpi	ER 9dpi
P-Value	0.050659	0.072491	0.0421489	0.0119054	0.0026542	9.02E-05	0.0069949	5.87E-07
([Chol][BTHCOO]_ToMV) vs ([Chol][BTHCOO])	CTR 4h	CTR 1dpi	CTR 5dpi	CTR 9dpi	ER 4h	ER 1dpi	ER 5dpi	ER 9dpi
P-Value	1.00E-08	0.0496913	0.0974411	0.0509317	0.0191976	0.0290281	1.00E-08	0.0443927
(BTHWA_ToMV) vs (BTHWA)	CTR 4h	CTR 1dpi	CTR 5dpi	CTR 9dpi	ER 4h	ER 1dpi	ER 5dpi	ER 9dpi
P-Value	1.40E-05	0.61438	1.02E-07	3.08E-07	0.0003793	0.0016682	0.1876423	0.1133478
([N4444][BTHCOO]_ToMV) vs ([N4444][BTHCOO])	CTR 4h	CTR 1dpi	CTR 5dpi	CTR 9dpi	ER 4h	ER 1dpi	ER 5dpi	ER 9dpi
P-Value	1.60E-08	2.71E-07	0.142576	1.13E-07	1.00E-08	3.40E-08	1.00E-08	1.00E-08
([N111010][BTHCOO]_ToMV) vs ([N111010][BTHCOO])	CTR 4h	CTR 1dpi	CTR 5dpi	CTR 9dpi	ER 4h	ER 1dpi	ER 5dpi	ER 9dpi
P-Value	1.03E-05	3.87E-05	0.0006633	1.00E-08	1.00E-08	1.00E-08	1.00E-08	1.00E-08

C)

(BTH_ToMV) vs (BTH)	Lox 4h	Lox 1dpi	Lox 5dpi	Lox 9dpi	JAR1 4h	JAR1 1dpi	JAR1 5dpi	JAR1 9dpi	PI II 4h	PI II 1dpi	PI II 5dpi	PII 9dpi
P-Value	0.2737283	0.0618111	0.0154143	1.00E-08	0.0010443	0.4811431	1.00E-08	1.00E-08	1.00E-08	1.00E-08	1.10E-08	1.00E-08
([Chol][BTHCOO]_ToMV) vs ([Chol][BTHCOO])	Lox 4h	Lox 1dpi	Lox 5dpi	Lox 9dpi	JAR1 4h	JAR1 1dpi	JAR1 5dpi	JAR1 9dpi	PI II 4h	PI II 1dpi	PI II 5dpi	PII 9dpi
P-Value	6.25E-05	1.00E-08	0.0570216	1.00E-08	0.0871674	1.00E-08	1.00E-08	0.1088466	0.0475104	1.00E-08	1.00E-08	1.00E-08
(BTHWA_ToMV) vs (BTHWA)	Lox 4h	Lox 1dpi	Lox 5dpi	Lox 9dpi	JAR1 4h	JAR1 1dpi	JAR1 5dpi	JAR1 9dpi	PI II 4h	PI II 1dpi	PI II 5dpi	PII 9dpi
P-Value	1.00E-08	1.00E-08	0.1039032	1.00E-08	1.00E-08	1.00E-08	1.00E-08	1.00E-08	1.00E-08	1.00E-08	4.54E-05	1.00E-08
([N4444][BTHCOO]_ToMV) vs ([N4444][BTHCOO])	Lox 4h	Lox 1dpi	Lox 5dpi	Lox 9dpi	JAR1 4h	JAR1 1dpi	JAR1 5dpi	JAR1 9dpi	PI II 4h	PI II 1dpi	PI II 5dpi	PII 9dpi
P-Value	3.70E-08	1.00E-08	1.00E-08	1.00E-08	0.0251255	1.00E-08	1.00E-08	1.20E-08	0.1714314	1.00E-08	0.0003552	1.00E-08
([N111010][BTHCOO]_ToMV) vs ([N111010][BTHCOO])	Lox 4h	Lox 1dpi	Lox 5dpi	Lox 9dpi	JAR1 4h	JAR1 1dpi	JAR1 5dpi	JAR1 9dpi	PI II 4h	PI II 1dpi	PI II 5dpi	PII 9dpi
P-Value	0.1742563	0.4637407	0.0001496	1.00E-08	1.00E-08	1.00E-08	1.00E-08	1.00E-08	0.0021768	1.14E-05	0.0853752	1.00E-08

Table S6. Results of gene expression analysis considering two independent variables (viral infection and inducer treatment) using a two-way ANOVA test. Red scores represent statistically significant results.

		4h									1dpi								
		CTR	ER	GluB	JAR1	Lox	NPR1	PAL	PI II	PR-1b	CTR	ER	GluB	JAR1	Lox	NPR1	PAL	PI II	PR-1b
BTH	MS	0,260	1,763	45,945	251,746	2,273	1,968	74,3599	5,4340	185,954	1,2609	22,952	112,841	278,152	72,757	40,859	15,9487	0,202	10,565
	F	0,339	0,9455	18,401	183,368	1,0749	1,1080	165,082	12,471	154,806	1,9104	22,470	59,953	306,799	47,691	38,441	37,556	0,341	10,367
	p	0,561914	0,333422	0,000044	0,000000	0,302561	0,295285	0,000000	0,000648	0,000000	0,170271	0,000008	0,000000	0,000000	0,000000	0,000000	0,000000	0,560655	0,001773
[Chol][BTHCOO]	MS	4,688	0,306	159,240	106,846	14,933	26,4691	48,1219	61,7604	39,846	0,6523	7,843	35,450	0,060	9,032	6,368	0,0000	3,424	3,189
	F	14,970	0,308	167,740	62,3329	7,2685	21,5011	114,240	62,2215	50,447	1,5139	10,525	19,719	0,071	6,778	6,846	0,000	4,842	7,800
	p	0,000204	0,580292	0,000000	0,000000	0,008345	0,000012	0,000000	0,000000	0,000000	0,221683	0,001642	0,000025	0,790572	0,010761	0,010386	0,991887	0,030276	0,006355
BTH-Wa	MS	8,212	2,529	21,201	206,646	28,710	3,3083	42,6715	463,585	11,272	0,0086	0,670	25,491	91,823	21,576	32,491	6,4799	35,770	7,102
	F	44,05	8,501	9,9036	1139,45	17,2269	3,0893	83,222	1107,872	21,207	0,0191	1,161	21,627	85,471	16,176	35,702	18,442	80,907	14,121
	p	0,000000	0,004458	0,002223	0,00	0,000074	0,082135	0,000000	0,000000	0,000013	0,890468	0,283979	0,000011	0,000000	0,000118	0,000000	0,000043	0,000000	0,000301
[N4444][BTHCOO]	MS	24,031	4,8241	215,095	181,429	25,052	7,4935	74,7512	49,8528	71,276	5,1539	20,981	131,613	33,238	7,498	20,470	0,2042	1,094	4,692
	F	67,760	11,389	156,473	313,381	13,4806	5,2360	140,311	68,131	52,808	11,5418	31,157	103,787	34,764	5,336	23,100	0,554	2,122	11,754
	p	0,000000	0,001083	0,000000	0,00	0,000404	0,024415	0,000000	0,000000	0,000000	0,001007	0,000000	0,000000	0,000000	0,023123	0,000006	0,458504	0,148607	0,000910
[N111010][BTHCOO]	MS	48,499	31,9012	269,698	317,570	0,813	11,642	38,6647	11,091	63,683	3,6616	36,285	266,644	91,703	85,321	47,987	6,5715	183,375	108,867
	F	36,986	48,102	182,648	520,770	0,4481	7,1702	37,0121	8,391	72,115	7,7606	64,831	221,325	100,867	64,955	51,946	13,854	337,777	170,212
	p	0,000000	0,000000	0,000000	0,000000	0,504909	0,008779	0,000000	0,004712	0,000000	0,006484	0,000000	0,000000	0,000000	0,000000	0,000000	0,000340	0,00	0,000000

		5dpi									9dpi								
		CTR	ER	GluB	JAR1	Lox	NPR1	PAL	PI II	PR-1b	CTR	ER	GluB	JAR1	Lox	NPR1	PAL	PI II	PR-1b
BTH	MS	3,8710	0,175	10,415	30,540	7,5693	0,4527	0,326	0,017	28,228	7,132	6,045	165,560	14,672	93,141	42,719	3,409	176,882	57,462
	F	7,8115	0,211	28,982	12,042	2,2365	0,828	0,1507	0,010	33,628	5,4864	4,593	207,617	11,964	115,259	61,841	1,6614	117,511	35,560
	p	0,006318	0,646844	0,000001	0,000794	0,138210	0,365311	0,698757	0,920414	0,000000	0,021322	0,034737	0,000000	0,000824	0,000000	0,000000	0,200641	0,000000	0,000000
[Chol][BTHCOO]	MS	0,2446	4,682	2,833	218,511	0,9356	0,0423	11,060	11,084	44,688	28,541	14,015	53,213	56,118	3,034	4,277	128,087	74,942	4,040
	F	0,802	6,980	6,347	132,151	0,3676	0,094	4,7152	17,180	74,162	20,500	10,654	131,07	35,631	2,918	8,923	66,757	96,943	2,673
	p	0,372926	0,009686	0,013481	0,000000	0,545824	0,760090	0,032470	0,000076	0,000000	0,000018	0,001543	0,000000	0,000000	0,090991	0,003610	0,000000	0,000000	0,105468
BTH-Wa	MS	9,2647	9,907	0,002	106,477	0,4284	0,1573	7,633	22,961	18,356	4,112	12,170	21,688	0,027	8,739	6,476	44,451	0,135	6,707
	F	32,668	6,537	0,004	71,322	0,1792	0,423	3,8963	27,261	26,165	3,825	10,160	49,87	0,025	11,340	12,315	25,810	0,124	5,637
	p	0,000000	0,012201	0,951863	0,000000	0,673082	0,516950	0,051391	0,000001	0,000002	0,053530	0,001961	0,000000	0,874065	0,001109	0,000698	0,000002	0,725787	0,019662
[N4444][BTHCOO]	MS	0,2463	8,425	8,535	9,570	14,2970	5,5949	0,610	25,061	45,796	50,037	48,593	63,677	23,085	2,966	0,289	78,123	165,375	2,806
	F	0,673	10,508	18,151	6,728	5,8649	9,4192	0,2775	27,797	72,131	43,083	44,467	150,75	19,202	3,551	0,387	40,1507	224,719	2,074
	p	0,414159	0,001656	0,000049	0,011042	0,017407	0,002822	0,599602	0,000001	0,000000	0,000000	0,000000	0,000000	0,000031	0,062685	0,535581	0,000000	0,000000	0,153253
[N111010][BTHCOO]	MS	0,7424	17,357	22,947	194,618	5,7428	4,0341	8,133	67,838	43,792	50,614	49,968	51,208	1,377	29,457	6,979	23,313	69,428	0,563
	F	1,6077	24,016	41,810	115,687	2,2577	7,847	3,7715	73,232	74,514	48,640	45,560	144,62	1,317	35,552	13,513	8,1892	65,513	0,388
	p	0,208010	0,000004	0,000000	0,000000	0,136377	0,006205	0,055192	0,000000	0,000000	0,000000	0,000000	0,000000	0,254126	0,000000	0,000398	0,005217	0,000000	0,534878

Table S7. Results of gene expression analysis considering three independent variables (viral infection, inducer treatment and time of harvest) using multivariate ANOVA. Red scores represent statistically significant interactions.

A)

BTH-Wa ET-pathway		CTR	ER
Virus*Inducer*Time	MS	6,885	8,410
	F	13,811	9,376
	p	0,000000	0,000005

BTH-Wa SA-pathway		GluB	NPR1	PAL	PR-1b
Virus*Inducer*Time	MS	20,68	5,026	19,700	14,42
	F	19,08	6,984	17,336	19,71
	p	0,000000	0,000140	0,000000	0,000000

BTH-Wa JA-pathway		JAR1	Lox	PI II
Virus*Inducer*Time	MS	119,86	18,260	129,73
	F	126,03	11,853	185,76
	p	0,000000	0,000000	0,000000

B)

[Chol][BTHCOO] ET-pathway		CTR	ER
Virus*Inducer*Time	MS	10,360	3,407
	F	16,975	3,658
	p	0,000000	0,012686

[Chol][BTHCOO] SA-pathway		GluB	NPR1	PAL	PR-1b
Virus*Inducer*Time	MS	69,579	4,816	43,867	16,02
	F	77,32	6,231	34,483	19,34
	p	0,000000	0,000389	0,000000	0,000000

[Chol][BTHCOO] JA-pathway		JAR1	Lox	PI II
Virus*Inducer*Time	MS	126,276	6,216	31,56
	F	87,238	3,566	40,49
	p	0,000000	0,014362	0,000000
